# Advances and prospects of orchid research and industrialization

**DOI:** 10.1093/hr/uhac220

**Published:** 2022-09-28

**Authors:** Diyang Zhang, Xue-Wei Zhao, Yuan-Yuan Li, Shi-Jie Ke, Wei-Lun Yin, Siren Lan, Zhong-Jian Liu

**Affiliations:** Key Laboratory of National Forestry and Grassland Administration for Orchid Conservation and Utilization at College of Landscape Architecture, Fujian Agriculture and Forestry University, Fuzhou 350002, China; Key Laboratory of National Forestry and Grassland Administration for Orchid Conservation and Utilization at College of Landscape Architecture, Fujian Agriculture and Forestry University, Fuzhou 350002, China; College of Forestry, Fujian Agriculture and Forestry University, Fuzhou 350002, China; Key Laboratory of National Forestry and Grassland Administration for Orchid Conservation and Utilization at College of Landscape Architecture, Fujian Agriculture and Forestry University, Fuzhou 350002, China; Key Laboratory of National Forestry and Grassland Administration for Orchid Conservation and Utilization at College of Landscape Architecture, Fujian Agriculture and Forestry University, Fuzhou 350002, China; College of Forestry, Fujian Agriculture and Forestry University, Fuzhou 350002, China; College of Biological Sciences and Technology, Beijing Forestry University, Beijing 100083, China; Key Laboratory of National Forestry and Grassland Administration for Orchid Conservation and Utilization at College of Landscape Architecture, Fujian Agriculture and Forestry University, Fuzhou 350002, China; Key Laboratory of National Forestry and Grassland Administration for Orchid Conservation and Utilization at College of Landscape Architecture, Fujian Agriculture and Forestry University, Fuzhou 350002, China

## Abstract

Orchidaceae is one of the largest, most diverse families in angiosperms with significant ecological and economical values. Orchids have long fascinated scientists by their complex life histories, exquisite floral morphology and pollination syndromes that exhibit exclusive specializations, more than any other plants on Earth. These intrinsic factors together with human influences also make it a keystone group in biodiversity conservation. The advent of sequencing technologies and transgenic techniques represents a quantum leap in orchid research, enabling molecular approaches to be employed to resolve the historically interesting puzzles in orchid basic and applied biology. To date, 16 different orchid genomes covering four subfamilies (Apostasioideae, Vanilloideae, Epidendroideae, and Orchidoideae) have been released. These genome projects have given rise to massive data that greatly empowers the studies pertaining to key innovations and evolutionary mechanisms for the breadth of orchid species. The extensive exploration of transcriptomics, comparative genomics, and recent advances in gene engineering have linked important traits of orchids with a multiplicity of gene families and their regulating networks, providing great potential for genetic enhancement and improvement. In this review, we summarize the progress and achievement in fundamental research and industrialized application of orchids with a particular focus on molecular tools, and make future prospects of orchid molecular breeding and post-genomic research, providing a comprehensive assemblage of state of the art knowledge in orchid research and industrialization.

## Introduction

With over 750 genera and 28 000 species, Orchidaceae constitutes one of the largest, species-rich families of flowering plants and has successfully colonized all continents except Antarctica [1, 2]. Orchids display extreme specializations particularly in the tropics, presenting several distinctive features like highly diversified flowers driven by orchid-pollinator interactions, dust-like, non-endospermic seeds, obligate association with mycorrhizal fungi, crassulacean acid metabolism (CAM) and unrivaled reproductive strategies contributing to a wide range of adaptation [3, 4]. Although exhibiting enormous diversity, the intrinsic nature of pollinator and mycorrhiza specification makes them particularly vulnerable to environmental change, and unsustainable harvest becomes a major additional risk. As a result, orchids now feature prominently among lists of threatened plant species, including all species in Appendices I and II of CITES [5] and 1601 species on the IUCN Global Red List [6]. Apart from their scientific fascination and significance, orchids are renowned for cut flowers and ornamental potted plants, accounting for a great part of the global floriculture trade [7]. Orchids also have long been commercialized as medicinal products and food [8], whereas breeding and mass production of commercially valuable orchids have long been hindered by a lack of scientific information on the mechanism involved in orchid growth and flower inducement.Therefore, from the time of Darwin to the present day, there was agreat deal of attention to this family in terms of its conservation, evolutionary relationship, and flower diversification. With thescientific tools now available for systematic research, there are emerging studies providing in-depth analysis addressing questions in orchids, with an increasing focus on comparative studies in the area of genomics. By identifying and validating functional genes, reconstructing phylogenetic relationships and performing genome-wide exploration, our understanding of the genetic basis of those unique characteristics of orchids has been fundamentallyreshaped. The availability of the wealth of genomic informationalso stimulates further studies on molecular breeding, new varieties selection and secondary component production that couldprotect and restore the wild orchids but also develop orchid industrialization in a way that satisfies the demand for commercialuses sustainably.

This review summarizes the prominent strategies and approaches utilized in orchid research including molecular markers, sequencing technologies, transgenic and gene editingtechniques, etc. with an enumeration of the studies leading to a major breakthrough in orchid biology such as orchid phylogeny,flower development and evolution of life forms. In addition, recent advances in biotechnology applied to the orchid industry together with future prospects pertaining to the post-genomic research, conservation, and utilization of orchids are briefly discussed.

## Orchid conservation

**Figure 1:**
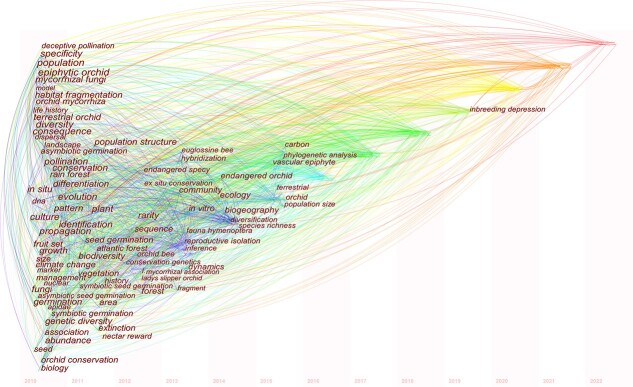
Knowledge mapping of orchid conservation*.* Visualization of keywords and their frequencies for orchid conservation by time series (2010–2022). Each node represents a keyword that first appears in the analysed data set. The published articles from 2010 to 2022 were retrieved from the Web of Science core collection.

Over the past 10 years, novel technologies have advanced our understanding of orchids and promoted revolution in orchid research. As modern molecular biology and computer science become more commonplace, the rapid development of genetic techniques has facilitated the unprecedented resolution of uncertainties in orchid biology. These techniques were heavily applied to studies with conservation purposes, including taxonomy, systematics, examination of genetic variation level, effective population size, and mating patterns; namely, population genetics [9]. To visualize the latest knowledge domain and emerging trends of orchid conservation-related literature, CiteSpace [10] was employed to conduct a scientometric investigation (Fig. 1). Figure 1 is generated by time series and depicting the keywords of the research on orchid conservation. It’s clear from the timelines that orchid conservation research has gradually shifted from descriptive theories and conventional practices to a high technology-based quantitative science, particularly in a direction toward molecular systematics. Notably, high-frequency keywords like ‘phylogenetic analysis’, ‘biogeography’ and ‘conservation genetics’ have occurred since 2013 (Fig. 1). Indeed, new molecular approaches have emerged in the past decades and will be the most powerful tool in providing new insights and understanding into orchid conservation.

### Orchid classification and systematics

Due to frequent hybridization and introgression, delineating taxonomic boundaries of orchids can be very difficult [11, 12]. The resulting uncertainties in the taxonomy and conservation status of orchids can largely hamper effective conservation strategy-making. Nowadays, markers include amplified fragments length polymorphism (AFLP), microsatellite DNA, chloroplast genomes, single nucleotide polymorphisms (SNPs), and restriction-site-associated DNA sequencing (RADseq) have been widely used in systematic and taxonomic studies [13]. These approaches are likely to be most important for delimitating species and intergeneric relationships. More than 10 new recorded genera or new genera with dozens of new species have been published with molecular evidence for their distinctiveness, including new genera *Danxiaorchis* [14], *Hsenhsua* [15], *Shizhenia* [16], and *Yunorchis* [17], the newly recorded genus *Thaia* [18] and genera with phylogenetic replacement, *Cymbilabia* and *Mengzia* [19, 20]. Newly discovered species with support from a phylogenetic tree based on nuclear and plastid DNA markers were found in several genera including *Bulbophyllum*, *Paphiopedilum*, *Gastrodia*, *Dendrobium*, *Liparis*, and *Cymbidium* [21–27]. Although challenges still remain, there have been great strides in the use of molecular tools for the description of new taxa, and the rapid development of new markers can be expected to further expedite the process of orchid systematic studies.

Along with the species-level identification, orchid phylogenetics have now employed next-generation sequencing (mostly Illumina techniques) for understanding higher-level relationships. Given its small size, uniparental inheritance, conservative gene content, and great numbers in cells, plastid genome (plastome) has so far been the most important source of data for plant phylogeny [28]. Since the first plastid genome has been reported in *Phalaenopsis aphrodite* subsp. *formosana* [29], so far there are 642 complete plastid genomes for Orchidaceae in Genbank (accessed 3 May 2022). Indeed, extensive studies have also been done in developing orchid plastid phylogenomics as a well-resolved, strongly supported, time-calibrated phylogeny is fundamental for circumscribing species, tribes and genera. Since the first DNA data-based phylogenetic classification of Orchidaceae was published [30], there has been a great deal of progress in resolving relationships and problematic placements at higher levels. We have seen an exponential growth in plastid phylogenomics to resolve and clarify relationships across orchids, including a supermatrix tree based on 75 chloroplast genes for 39 species covering all orchid subfamilies 16 of 17 tribes [31]; reconstruction of phylogeny and temporal evolution of Orchidaceae based on 76 and 38 coding genes of plastid and mitochondrial genomes, respectively [32]; a first-time phylogenetic placement for Codonorchideae (Orchidoideae), Podochilieae and Collabieae (Epidendroideae) based on 78 plastid coding genes [33]; plastid phylogenomic resolution of subtribes Aeridinae [19] and Goodyerinae [34]; and genera phylogenetics such as *Dendrobium* [35], *Cymbidium* [36], *Holcoglossum* [37] and *Paphiopedilum* [38], suggesting plastome has been a mainstay for addressing finer-scale phylogenetic questions in orchid evolutionary studies.

In parallel with the examination of the plant’s organelle genomes, opportunities are rising for using transcriptomic data for orchid phylogenetics. Several orchid phylotranscriptomics have been performed for addressing either broad-scale or shallow-scale orchid relationships including species diversification and genome evolution, as well as key traits like sexual deception [39–43]. These studies also give insights beyond phylogenetic analysis to uncover the morphological evolutionary histories of thousands of unlinked genes. Therefore, repurposing transcriptomic studies provides an additional approach and perspectives for orchid systematic studies. Collectively, drawing on these informative data for orchid classification and systematics has gained important insights into the mechanisms driving the extraordinary species diversity and served as an invaluable resource for conservation implications.

### Population genetics

At the population level, identifying species that should be treated as a high priority for conservation is also struggling for conservation planning [44], thus highlighting the need for population genetic analysis. In orchids, unique traits like deceptive pollination and dust-like, wind-dispersed seeds have promoted gene flow between populations [45]. These reproductive strategies have led to significant heterogeneity in the genetic structure among orchid populations [46]. To investigate the population genetics of endangered orchids, marker-assisted approaches have been widely employed in the assessment of genetic diversity, genetic drift, level of inbreeding, and gaining insights into contemporary and historical dispersal for populations identified as being at risk. Genetic diversity studies for the populations of two critically endangered *Amitostigma* species were conducted to identify which entities to preferentially conserve and determine optimal conservation strategy [47, 48]. Studies on *Cypripedium* showed random genetic drift and limited gene flow have a great impact on its genetic diversity and population structure [49, 50], while distinct levels of genetic diversity occur in *Cypripedium* populations with different types of habitats and climates [51, 52]. The accumulation of ancestral variation and genetic admixture from multiple post-glacial colonization routes may explain the high genetic diversity of *Cypripedium* in central Europe, enabling the long-term stability of the species in different biogeographical regions [53, 54]. Anthropogenic disturbance accounts for the major factors driving the orchid habitat fragmentation and deterioration, especially for those thriving as epiphytes [55]. Population genetic structure analysis of an epiphytic orchid, *Bulbophyllum occultum* showed self-pollination and genetic drift have contributed to its high population genetic and phenotypic differentiation, despite it being impacted severely by deforestation [56]. In another case of a dominant clonal *B. bicolor*, low genotypic diversity and lack of spatial genetic structure led by skewed clonal reproduction have contributed to the loss of sex and extinction debt, which demands urgent conservation attention such as *ex situ* collection [57]. Orchids have specific adaptations to pollinators that could promote crossing and avoid inbreeding depression [58]. Several studies have investigated the relative fitness of the population resulting from selfing and outcrossing by using molecular markers. Genetic diversity and structure of *Cattleya* were evaluated by ISSR markers and the results showed that *Cattleya* populations undergo a drastic population decline while deceptive pollination and long-distance seed dispersal may help with higher genetic variability [59, 60]. Blambert *et al*. [61] reported the selfing rates and levels of genetic diversity for two closely related *Jumellea* species with different reproductive systems. The results showed that the selfing rate and magnitude of inbreeding depression are negatively correlated. A study that examined the population genetic patterns of the populations of *Platanthera praeclara* indicated that its small population size could lead to severe inbreeding depression [62]. Genetic diversity and population structure of *Calibrachoa* showed that species presenting inbreeding are more likely to suffer from a loss of alleles, resulting in a low level of population structure and diversity [63]. In a case that used double-digest restriction-site associated DNA (ddRAD) sequencing to evaluate the conservation status of three threatened species of *Corybas*, the gene structure results provided evidence for hybridization and introgression within the *Corybas* complex, thus leading to blurred taxonomic boundaries [64].

Taken together, the reliability and efficiency of molecular tools have provided more opportunities for examining a wider spectrum of orchid population genetics, facilitating the development of genetic studies on more endangered orchids and the possibility of answering unresolved biological questions, thereafter guiding the conservation practices.

### Orchid biology

#### Deceptive pollination

Orchids have evolved to diverse pollination mechanisms with an overrepresentation of floral mimicry (deceptive pollination) compared to other plants [65]. For a long time, ecological investigations were mostly adopted to understand the driving forces of orchid deception, while the knowledge gap remains to be filled between the underlying mechanisms of the evolution of mimicry and species diversification [66]. Several studies have performed novel and multidisciplinary approaches to provide new insights into this research area. For example, genus phylogeny suggested that the unidirectional transition in nectar presence/absence is associated with food deception, which is an ‘evolutionarily stable strategy’ in *Epidendrum* [67]. A study incorporated field experiments and chemical findings into phylogenetic analysis concluded that the sexually deceptive orchid *Chiloglottis* undergoes pollinator-driven speciation [68]. A similar attempt was reported in genera *Serapias* and *Iris* to provide evidence for evolutionary transitions between two different pollination strategies [69]. *Stearoyl-acyl carrier protein desaturase* (*SAD*), the first gene involved in the biosynthesis of semiochemicals for sexual deception was found and it’s responsible for alkene difference between *Ophrys* species and pollinator-driven ‘genic’ speciation [70]. Despite the recent advances in our understanding of the orchid deception, it is evident that much remains to be learnt. In light of the methodological progress, our capability will be greatly enhanced to examine the basis of pollination deception at an unparalleled depth.

#### Mycorrhizal associations

A common feature of orchids is their obligate mycorrhizal associations, which play crucial roles in orchid life cycle and population dynamics [71, 72]. Identification of orchid mycorrhizal fungi (OMF) has been extensively studied in at least 200 genera [73]. Most OMF belongs to three Basidiomycota lineages, namely Ceratobasidiaceae, Tulasnellaceae, and Serendipitaceae [74]. Moreover, orchid distribution is considered associated with OMF diversity [75], which is jointly regulated by environmental factors, spatiotemporal variations, biogeography, and the phylogenetic constraints of host orchids [72]. Multifaceted evidence from radiocarbon (∆^14^C), molecular markers and genomic data suggested that both fully mycoheterotrophic and partial mycoheterotrophic orchids obtain carbon from fungi [76, 77]. And the concept that OMF receives nothing in return has been overturned by a study that examined the expression of fungal and plant nitrogen (N) transport and assimilation genes, the results showed mycoheterotrophic orchid and its fungal partner have a mutualistic association [78]. Whereas disentangling the factors determining OMF specificity and molecular mechanisms of this symbiosis still represents a major challenge. Nevertheless, a broader investigation of OMF is needed to translate more orchid-fungi crosstalk, and the availability of fungal reference genomes [79] is expected to enable metagenomic studies to be implemented to address these questions. These studies will be especially important for the regeneration of rare and endangered orchids that link orchids and OMFs in efforts to inform conservation.

#### Crassulacean acid metabolism

CAM is a key innovation for orchids, representing a specialized drought-adapted photosynthetic pathway that enables plants to grow in the arid environment [80]. Several transcriptomic studies have been done to illuminate the origin and evolution of CAM pathway. PEPC is a key enzyme in CAM pathway, phylogenetic analysis of the PEPC family based on transcriptome data revealed that orchid *PEPC* genes originated from the ancient duplication of monocots, with CAM developed earlier than C4 [81]. Zhang *et al.* [82] reported a comprehensive comparison of carbon fixation pathway genes based on transcriptomic data of 13 orchid species, and revealed that CAM may have evolved primarily by changes in the transcription level of key carbon fixation pathway genes. Differentially expressed genes between circadian day and night were identified in *Pha. equestris*; these genes were mostly enriched in carbon fixation, circadian clock regulation, photosynthesis, and signal transduction pathways [83]. Other studies that employed physiological and biochemical measures to examine the diel dynamics of carbon gain and metabolites of CAM have provided more opportunities to study orchids’ adaptation to dry environments, especially in response to projected global warming [84–86].

## Whole genome sequencing

**Figure 2:**
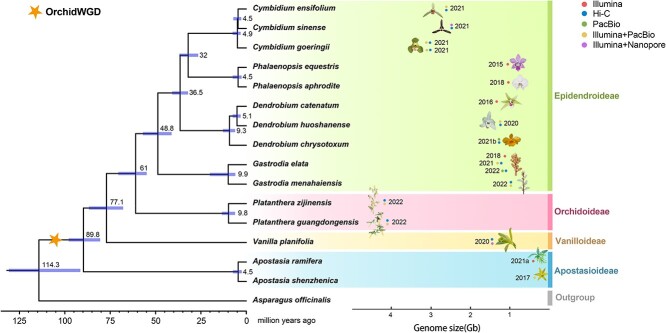
Phylogenetic relationship and divergence time of 15 published orchid genomes. Maximum likelihood phylogenetic tree was constructed using single-copy orthologous sequences from 15 published orchids covering four subfamilies, with *Asparagus officinalis* as an outgroup. Divergence time was calculated by MCMCTree in PAML v.4.9 package. The orange star indicates the lineage-specific WGD (Orchid WGD) event for Orchidaceae. Subfamilies are listed to the right of the tree. Sequencing techniques for each study were marked in dots with different colors.

With the advent of sequencing techniques, we have witnessed a paradigm shift from microarray-based genotyping studies to whole genome sequencing. The adoption of third-generation sequencing approaches that generate longer reads (e.g. Pacific Biosciences HiFi [87], Pacific Biosciences SMRT [88], Oxford Nanopore [89]) and methods for chromosome conformation capture (e.g. Hi-C) have ushered the revolution of higher quality sequencing and assembly in genome contiguity and accuracy of plants, even for orchids that have large and complex genomes. To date, 16 orchid genomes encompassing four subfamilies (Apostasioideae, Vanilloideae, Epidendroideae, and Orchidoideae) have been published with some of them having updated versions of assemblies and annotations (Fig. 2**;**Table 1). These genome studies together with high-quality referenced transcriptomic data have given rise to major conceptual or technical breakthroughs of orchid research and overturned many classical views of orchid biology.

**Table 1:** Whole genome sequences of reported orchids species.

$\includegraphics{\bwartpath uhac220t1}$

a A, haplotype-A, B, haplotype-B

*Pha. equestris* was the first reported orchid genome that contributed to a greater understanding of orchid morphological evolution and physiological adaptation [3]. The highly sophisticated floral structures, epiphytism and CAM were considered pivotal traits that jointly promoted the rapid radiation of orchids. A chromosome level assembly of *Pha. aphrodite* showed that lineage-specific expansion of FRS-like subclade might be an adaptation to the unstable light condition that is associated with epiphytes [90]. The genome sequence of primitive orchid *Apostasia shenzhenica* represents a milestone in research on orchid genomics [91]. By reconstructing an ancestral orchid gene toolkit and evolutionary history of orchids within the angiosperms, together with the exhausted investigation on *Apo. shenzhenica* genome characteristics and transcriptome data covering all orchid subfamilies, this study provided significant insights into key innovations, origins, adaptation and diversification of orchids. Absolute dating of whole-genome duplication (WGD) event in *Apo. shenzhenica* showed a lineage-specific WGD in orchids which occurred shortly before the divergence of five subfamilies. Another interesting feature in orchids is that all orchids showed mycorrhizal fungi association (partial mycoheterotrophy) during seed germination and the early stage of protocorms [92]. Genomes of obligate mycoheterotrophic orchids were reported in two species in *Gastrodia* [93–95] and *Platanthera guangdongensis* [77]. Both studies revealed contracted plastid genomes and substantial loss of genes involved in photosynthesis of fully mycoheterotrophic orchids, while elevated expression of trehalase could be a critical adaptation for mycoheterotrophy, enabling orchids to hijack trehalose from fungi and resynthesize it as sucrose for internal use [77].

For thousands of years, the active compounds in orchids have been used in traditional medicine or as health food supplements. These ancient remedies have now been scientifically scrutinized at the molecular level. *Dendrobium* is the second largest genus in Orchidaceae and is renowned for medicinal use in Asia. Expansion of resistance-related genes and highly expressed polysaccharide synthase gene families in the *Dendrobium* genome suggested a powerful immune system and high tolerance to environmental stress that contributes to its wide distribution [96]. An updated version of the chromosome-level *Dendrobium* genome combined with genome-wide association studies (GWAS) revealed several genes associated with the biosynthesis of active ingredients and stem production in *Dendrobium* species [97]. Another high-quality chromosome level *Dendrobium* genome, *D. chrysotoxum* provided important insights into the interplay among carotenoid, abscisic acid (ABA), and ethylene biosynthesis, demonstrating the regulatory mechanisms of the relatively short flowering period of yellow-flower orchids [98]. The first haplotype-resolved genome assembly using Pacbio HiFi sequencing of orchid is completed in *Bletilla striata*, a traditional medicinal herb in China [99]. The study reconstructed the ancestral karyotype (18 chromosomes) of seven orchids and functionally verified the key transcription factor (MYB) involved in polysaccharide biosynthesis, laying a foundation for molecular-assisted breeding for orchids with high medicinal values. Vanilla, a stunning spice that is mostly derived from *Vanilla planifolia*’s pods, is now ubiquitous in worldwide food and beverage production. A chromosome-scale genome assembly of *V. planifolia* was reported to provide a better understanding of the genes associated with vanillin pathway, which will enable accelerated breeding of vanilla pods with higher quality and productivity [100]. The other chromosome-level, haplotype-phased *V. planifolia* genome based on Pacbio HiFi sequencing was completed to address the partial endoreplication of *V. planifolia* and take a step forward to further elucidate this complex genome [101].

Orchids exhibit specialized and complex floral structures that led to marvelous species richness; these unique traits play an instrumental role in the course of orchid evolution*.* A comprehensive inspection of floral shape, color, and scent has been conducted in *Cymbidium*, a genus with vital commercial importance in the world floriculture industry*.* Three *Cymbidium* genomes (*C. ensifolium*, *C. sinense*, and *C. goeringii*) unveiled the important genetic clues to phenotypic traits such as colorful leaves, diversified flowers, and fragrance, which are primarily regulated by MADS-box, MYB, and TPS gene families, respectively [102–104]. Furthermore, changes in gene number and gene expression can affect flower morphogenesis by altering floral structure and color that foster a variety of mutants, providing great potentials for orchid molecular breeding and floriculture.

In a nutshell, new sequencing technologies are producing genome assemblies of increasing quality, deciphering orchid genomes can provide a comprehensive catalog of genomic information that could empower the studies of important traits and evolutionary mechanisms for the breadth of species with significant ecological and evolutionary importance.

## Orchid database

The emergence and availability of massive sequence data have opened new interfaces with computer science, allowing the establishment of multiple orchid databases. OrchidBase was established in 2011 and has now updated to version 5.0, which accommodates whole-genome sequencing and transcriptomic data for *Apo. shenzhenica*, *D. catenatum* and *Pha. equestris*, and floral transcriptomic sequences from 10 orchid species covering all five subfamilies [105] (http://orchidbase.itps.ncku.edu.tw/). Orchidstra 2.0 [106] (http://orchidstra2.abrc.sinica.edu.tw) is another orchid database for transcriptomics resource that includes orchid transcriptome assembly and gene annotations of 18 orchid species belonging to 12 genera across five subfamilies. Other databases like OOGB (http://predictor.nchu.edu.tw/oogb) and PhalDB [107] also provided quantities of sequence information about the genome, transcriptome, and miRNA data of *Oncidium* and *Phalaenopsis*, respectively. These datasets provide the capacity and platform for rapid data mining, generation, and analysis. The aggregation and navigation of different types of orchid data enable the broader biology community to access orchid bioinformatics and perform genome-wide analysis with a lower level of computational skills.

## Genome-wide studies

On the basis of large-scale whole-genome data, genome-wide identification and comparative study of a multiplicity of gene families have been conducted in several orchids. Recent publications have reported gene families like YABBY, terpene synthase (TPS), R2R3-MYB, KNOTTED1-like homeobox (KNOX), WRKY, autophagy (ATGs), and MADS-box [108–115] and their expression analysis in *Dendrobium*, *Cymbidium*, and *Phalaenopsis*. Phylogenetics, physicochemical properties, and comparative transcriptomics were performed to identify key genetic basis and molecular mechanisms determining the organogenesis and morphogenesis of orchids. Also, from a perspective of floriculture development, most economically important traits are usually inherited in a quantitative manner such as floral scent and color. Therefore, genome-wide associate studies can contribute to the discovery of candidate genes associated with these important traits. Genotyping-by-sequencing (GBS) approach based on Illumina sequencing has been applied to study four sexually deceptive orchids of *Ophrys*. Highly differentiated polymorphisms were found in genes that are involved in floral scent, including several SNPs linked to pseudo-pheromones related genes [116]. GWAS was performed by combining SNPs and floral aesthetic traits to identify QTL that is associated with color-related traits in *Phalaenopsis* [117]. The results indicated 10 quantitative trait loci and 35 candidate genes that were associated with color-related traits and anthocyanin biosynthesis, respectively, providing important selection markers for *Phalaenopsis* breeding.

## Genes associated with important traits

**Figure 3:**
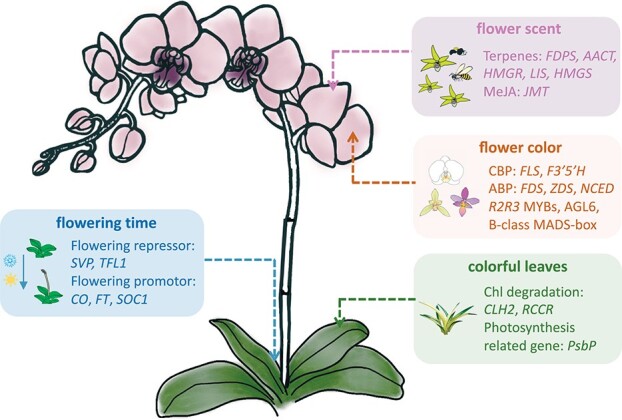
Genes related to key horticulture traits of orchids. Genes involved in floral scent biosynthesis include *FDPS*, *AACT*, *HMGR*, *LIS*, *HMGS*, and *JMT* in methyl jasmonate (MeJA) biosynthesis. Genes regulating chlorophyll degradation such as *CLH2* and *RCCR*, and photosynthesis-related genes *PsbP* are responsible for orchid colorful leaves. Flower color is mainly determined by genes involved in carotenoid biosynthesis (CBP) and anthocyanin biosynthesis pathway (ABP), as well as TFs like R2R3 MYBs, AGL6, and B-class MADS-box. The transition from vegetative development to reproductive growth requires the mediation of multiple flowering repressors (*SVP*-like, *TFL1*-like genes) and promotors (*CO*-like, *FT*-like, *SOC1*-like genes).

The development of whole genome sequencing, *de novo* transcriptome and molecular toolkit for functional analysis have allowed identification, quantitative comparison and functional characterization of genes related to important traits in orchids. Key genes and transcription factors (TFs) involved in floral pattern, flowering time, coloration, floral scent, colorful leaves, and other important properties were identified and validated (Fig. 3). These studies provide nearly unlimited possibilities in orchid research that could translate complex orchid biology to quantitative information and lay a solid foundation for orchid molecular breeding.

### Floral patterning

**Figure 4:**
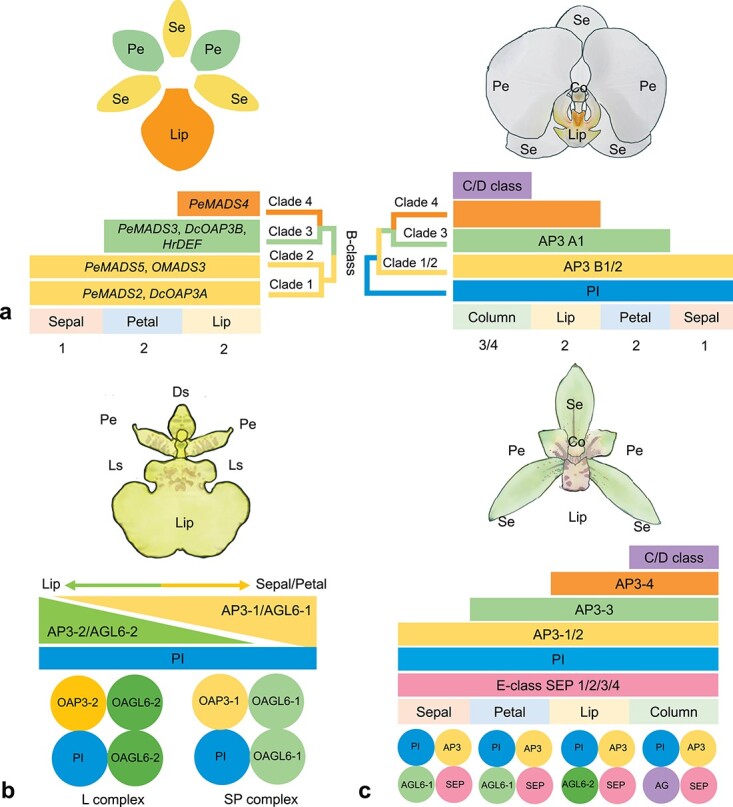
The proposed ‘Orchid Code’, ‘Homeotic Orchid Tepal’ (HOT) model, ‘Perianth code’ (P code) and *Cymbidium ensifolium*’s MADS-box model for flower development of orchids*.***a** Orchid code (left) and HOT model (right). In ‘orchid code’, clades 1–4 of B-class *DEF/AP3*-like genes specify the orchid perianth formation, upon which ‘HOT model’ proposed that *PI* and *AP3B* clades of B-class MADS-box genes determine all four whorls of flower identity. The joint contribution of *PI* and both *AP3A1* and *AP3Bs* regulate the formation of lateral petals. Both *PI* and *AP3* clade genes are involved in the formation of the lip. C- and D-class MADS-box genes specify column development. **b** Perianth code based on *Oncidium*. For ‘P code’, *PI* is expressed in lip, sepal and petal and interacts with different AP3-like and AGL-like proteins leading to lip or sepal/petal development. OPI combines with OAP3–2 and two OAGL6–2 to form L complex (Lip program) that determines lip development. SP complex (Sepal/Petal program) is composed of OPI, OAP3–1, and two OAGL6–1, regulating sepal and petal development. **c** MADS-box model of *Cymbidium ensifolium*. In *C. ensifolium*, E-class *SEP*-like and B-class *PI* and *AP3*-like genes are expressed in all flora whorls with C-and D- class genes expressed in column. Co, column; Ds, dorsal sepal; Ls, lateral sepal; Pe, petal; Se, sepal. Numbers 1, 2, 3/4 represent flower whorls.

Floral homeotic genes that encode MADS-box transcription factors play crucial roles in flower development, of which type II MADS-box genes are renowned for their roles in the specification of floral organ identity [118]. Most orchids display a zygomorphic flower with a distinguished lip in the second whorl, this particular floral architecture presents an exciting opportunity to examine the classical ABCDE model of flower development. Since the ‘Orchid Code model’ [119], ‘HOT model’ [120] and ‘Perianth code’ (P code) [121] have been proposed, a wide-ranging exploration of transcriptome and expression profiles of *Cymbidium* species and their mutants has been conducted to investigate the flower formation of orchids [122–125]. The results from the transcriptomic based analysis were further verified by whole genome data of *C. ensifolium* [104], *C. sinense* [102] and *C. goeringii* [103], which suggested that B- and E- class MADS genes play fundamental and dualistic roles in determining perianth formation while C- and D- class are involved in carpel and gynostemium (column) development (Fig. 4). To better understand the characteristics of ABCDE genes among different lineages, we identified and classified the MADS-box genes for all available orchid genomes (Table 2). Gene duplication in type II MADS genes seems common in orchids [3, 104] and most ABCDE clades look similar in gene numbers between closely related species, while some of them have extremely higher or lower copies (e.g. Bs in *C. ensifolium* and C/D in *G. elata*). In *Apostasia*, fewer B-AP3 and E class genes are considered to form an undifferentiated lip and partially fused column, making it an actinomorphic feature [91], whereas due to the dearth of knowledge pertaining to orchid flower development, the evolutionary buildup of gain and loss of ABCDE genes remains to be investigated. Notwithstanding, an increasing number of functional studies have been done to elucidate the function of these floral organ identity genes. Ectopic expression and virus-induced gene silencing (VIGS) were performed to investigate *SEPALLATA* (*SEP*) genes in determining floral organ identity of *Phalaenopsis* [126]. The model of perianth formation in orchids has been validated by the suppression of L complex activity in lips in *Oncidium* and *Phalaenopsis*, demonstrating *AGAMOUS-LIKE6* (*AGL6*)-like MADS-box genes are exclusively required for lip development, a specialized petal for most orchids [121]. Ectopic expression of *PeMADS28*, a Bs gene of MADS-box gene family in *Arabidopsis* can lead to abnormal ovule development, indicating the conserved function of Bs in ovule integument development [127]. The transgenic *Dendrobium* was generated for elucidating the functions of C- and D-class MADS-Box genes, in which two *AGAMOUS* (*AG*) genes, *DOAG1* and *DOAG2* were downregulated by microRNA interference [128]. The result showed that both genes exert different roles in specifying reproductive organ identity, *DOAG1* affects floral meristem determinacy and floral organ development, while *DOAG2* regulates perianth and gynostemium (column) development. A study based on VIGS and transient overexpression of *Pha. equestris* revealed that two *DROOPING LEAF/CRABS CLAW* (*DL*/*CRC*) genes play an important role in the innovation of orchid reproductive organs [129]. Transcriptome analysis combined with yeast two-hybrid assays of a greenish flower mutant of *Habenaria radiata* showed that this phenotype is caused by loss of function of an E-class MADS-box gene (*HrSEP-1*), which plays an important role in column, lip, and petal development [130]. In addition to MADS-box genes, it was also found that overexpression of miR390, a miRNA involves in diverse processes of plant growth and development from *C. goeringii* to transgenic *Arabidopsis* can alter normal reproductive organ development [131]. Valoroso *et al.* [132] found that *DIV*, *RAD*, and *DRIF* in the MYB gene family might be involved in the establishment of flower bilateral symmetry of *Orchis italica*, an orchid with resupinate flowers. Other regulatory mechanism includes the coordinated signaling of phytohormones such as ethylene, auxin, GA, and ABA also play important roles in orchid floral organ development [133,134]. All these studies specify that the diverse roles of MADS-box genes and other floral identify regulators have contributed to the specialized flower morphogenesis of orchids.

**Table 2:** Numbers and categories of MADS-box genes in published orchid genomes.

**Category**	***Apostasia shenzhenica***	***Apostasia ramifera***	***Vanilla planifolia***	***Platanthera zijinensis***	***Platanthera guangdongensis***	***Gastrodia elata***	***Phalaenopsis equestris***	***Phalaenopsis aphrodite***	***Dendrobium catenatum***	***Dendrobium chrysotoxum***	***Cymbidium ensifolium***	***Cymbidium goeringii***	***Cymbidium sinense***
Type II (Total)	27	23	39	29	27	27	29	45	35	32	38	44	42
MIKCc	25	22	36	27	25	27	28	34	32	28	34	34	41
A	2	2	6	3	3	3	3	3	4	4	4	4	6
Bs	1	1	1	1	1	2	1	1	2	4	7	1	6
B-PI	1	1	1	2	2	3	1	1	1	2	1	1	1
B-AP3	2	2	3	4	4	2	4	4	4	3	4	4	4
C/D	4	3	5	5	5	1	5	7	4	4	4	4	4
E	3	3	2	3	3	3	6	6	5	5	4	6	4
AGL12	1	1	0	1	0	0	0	0	0	1	0	0	0
SOC1	2	0	2	1	1	4	2	2	2	1	3	4	4
SVP	2	3	5	1	1	0	1	2	3	1	2	4	3
ANR1	4	4	4	2	1	4	2	2	3	1	1	1	2
OSMADS32	1	0	3	1	1	0	0	1	1	1	1	1	1
AGL6	2	1	3	3	3	5	3	5	3	1	3	4	6
FLC	0	0	0	0	0	0	0	0	0	0	0	0	0
AGL15	0	1	1	0	0	0	0	0	0	0	0	0	0
MIKC^*^	2	1	2	2	2	0	1	3	3	4	4	6	1
Type I (Total)	9	7	16	14	16	13	22	9	28	26	33	30	53
Mα	5	5	12	11	14	8	10	3	15	19	27	26	28
Mβ	0	0	0	0	0	0	0	2	0	0	0	0	17
Mγ	4	2	4	3	2	5	12	4	13	7	6	4	8
**Total**	**36**	**30**	**55**	**43**	**43**	**40**	**51**	**54**	**63**	**58**	**71**	**74**	**95**

### Floral color

Anthocyanin and carotenoid are major pigments that depict flower colors and key TFs such as MYBs play prominent roles in their biosynthesis [135]. In *Phalaenopsis* cultivars, the differential expression of *PeMYB2*, *PeMYB11*, *PeMYB12* was concomitant with complicated floral pigmentation patterning [136]. Transient overexpression of these *R2R3-MYBs* in *Phalaenopsis* verified that they can promote anthocyanin accumulation, and these *PeMYBs* could activate the expression of downstream structural genes. VIGS and transient overexpression of PeMYB4L, a member of R2R3-MYB verified its role in regulating anthocyanin biosynthesis in *Phalaenopsis* [137]. In *Cattleya*, transient overexpression of its two *R2R3-MYBs* (*RcPCP* and *RcPAP*) in a *Phalaenopsis* hybrid showed elevated expression of structural genes involved in carotenoid biosynthesis and anthocyanin biosynthesis, respectively, resulting in yellow or red pigmentation in *Phalaenopsis*’s perianth [138]. The insertion of a retrotransposon, *Harlequin Orchid RetroTransposon 1* (*HORT1*) in *PeMYB11* enhanced its expression and resulted in high accumulation of anthocyanins in harlequin flower [139]. 

It’s also widely acknowledged that MYB–basic-helix–loop–helix (bHLH)–WD40 repeat (WDR) (MBW) ternary complex are key regulators determining floral pigmentation and patterning [140]. Comparative transcriptomics of *Pleione* showed a MBW protein complex formed by *PlMYB10*, *PlbHLH20*, or *PlbHLH26* and *PlWD40–1* can repress the expression of *flavonol synthese* (*FLS*), the key structure gene in the anthocyanin biosynthesis pathway that contributes to color formation, resulting in color variations in wild populations [141]. Transient overexpression of a bHLH protein PeMYC4 plus a R2R3 MYB PeMYB4L showed a negative regulation of the anthocyanin accumulation by inhibiting the expression of structure gene *chalcone synthase* (*CHS*) [137]. Characterization of the structure genes involved in the anthocyanin biosynthesis has also been extensively studied in orchids. Metabolite profiling and transcriptomic analysis of sexually deceptive *Chiloglottis* showed downregulation and upregulation of *FLS* at young and mature bud stages, respectively, contributing to the stark color contrast between callus and lip, which enhances the visibility of the mimicry [142]. In *C. kanran*, transient expression of three structure genes *CHS*, *dihydroflavonol reductase* (*DFR*), and *anthocyanidin synthase* (*ANS*) caused purple-red pigmentation in white *C. kanran* flower [143]. For *Paphiopedilum hirsutissimum*, two structure genes (*F3H* and *CHS*) involved in anthocyanin biosynthesis and major carotenoid biosynthesis genes including *VDE*, *NCED*, and *ABA2* were strongly downregulated in albino phenotype compared to normal flowers, suggesting the color variation of *Pap. hirsutissimum* is a result of changes in anthocyanin and carotenoid contents [144]. Anthocyanin biosynthesis is also modulated by several phytohormones including ethylene, cytokinins, and ABA [145–147]. In orchids, two transcriptome-based analysis in *Dendrobium* showed auxin, ABA, and ethylene play various modulating roles in flower color formation [148,149]. Whereas the direct association between color formation and phytohormones, in the presence of other regulators that lead to the activation of anthocyanin biosynthesis remains unknown in orchids. Apart from MYB TFs, on the basis of P code in orchids, Hsu *et al.* [121] found that Class B- and *AGL6* MADS-box genes have additional functions than determining floral identifies, in which B and AGL6 proteins form L (OAP3–2/OAGL6–2/OPI) and SP (OAP3–1/OAGL6–1/OPI) complexes that regulate pigmentation of *Phalaenopsis*’s perianth. Also, *AP3*/*AGL6* genes may act together with *RcPAP1/2*, *RcPCP1* in shaping the spatiotemporal pattern of *Cattleya*’s color formation, further supporting the MADS-box genes’ potential role in regulating color differentiation of flower segments [138].

### Floral scent

Floral scent is one of the most important signals to attract pollinators, especially for some sexually and food deceptive orchids [150, 151]. On the other hand, fragrant orchids are also coveted commodities in the global orchid market. Studies on orchid floral scents have mainly focused on the characterization of genes encoding the key enzymes responsible for the synthesis of volatile compounds such as terpenes and methyl jasmonate (MeJA) [152]. Volatile terpenoid metabolism genes including *farnesyl diphosphate synthase* (*FDPS*), *acetyl-CoA C-acetyltransferase* (*AACT*), *hydroxymethylglutaryl-CoA reductase* (*HMGR*), linalool synthase (*LIS*), and 3-hydroxy-3-methylglutaryl-CoA synthase (*HMGS*) were found to be responsible for floral scent of *C. goeringii* [103, 153]. In *C. faberi*, *jasmonic acid carboxyl methyltransferase* (*JMT*), an important gene in MeJA biosynthetic pathways was highly upregulated in blooming flowers [154]. Similarly, MeJA biosynthesis-related genes were found exhibited maximal expression levels in opened flowers of *C. ensifolium* [155]. In terms of floral scent regulation, CIRCADIAN CLOCK ASSOCIATED1 (CCA1) TF was reported to regulate diurnal emission of floral scent in *Oncidium* [156] and diurnal scent emission of *Pha. violacea* was positively regulated by circadian clock and light factors which are associated with structural genes and TFs involved in monoterpene biosynthesis [157]. Functional characterization of orchid floral scent has been investigated in *Dendrobium*, *Phalaenopsis*, and *Cymbidium*. In *D. officinale*, genes involved in geraniol synthase (GES) were transiently expressed in *Nicotiana benthamiana*’s leaves, resulting in the accumulation of geraniol *in vivo* [158]. Functional analysis of several TFs (BHLH, Ethylene Response Factor (ERF), NAM, ATAF, and CUC (NAC)) and *TPS* genes involved in monoterpene biosynthesis was performed for *Pha. bellina*, indicating they can induce monoterpene production in scentless orchids [159, 160]. *TPS* genes of *C. faberi* have also been ectopically expressed in *Escherichia coli*, with β-myrcene, geraniol, and α-pinene as the main product catalyzed by *CfTPS18* [112]. Still, the type, regulation, and function of volatile compounds biosynthesis require further studies in orchids. Further progress in transcriptomic and functional studies may lead to more solid findings for revealing the molecular basis of orchid floral scent.

### Colorful leaf

Colorful leaf is another ornamental character for orchids. Knowledge of the underlying mechanisms that give rise to colorful leaves could help with the effective selection of desirable leaf traits. Leaf color is primarily determined by pigments such as chlorophyll and anthocyanin, mutation or different expression of genes associated with their biosynthesis and metabolism may lead to leaf color variations [161]. Differential expression of genes involved in the chlorophyll biosynthesis and degradation was found responsible for yellow- or silver-leaf phenotype in *Cymbidium* [104, 162, 163]. Comparative transcriptomic analysis between normal and yellow color-mutant leaves of *C. sinense* ‘Dharma’ showed that *chlorophyllase* (*CHL2*) and *red Chl catabolite reductase* (*RCCR*) which are key genes involved in chlorophyll degradation were highly expressed in the yellow-leaf mutant [164]. In *C. sinense* ‘Red Sun’, metabolic changes during the red to green leaf color transition were qualitatively and quantitatively analysed [165]. UPLC-MS/MS and PCR results showed that decreasing levels in 15 metabolites associated with anthocyanins biosynthesis, together with the down-regulation of genes encoding anthocyanin synthesis contribute to the leaf variegation of *C. sinense* ‘Red Sun’. Purple leaves mutant of *D. biggibum* leaves is considered associated with the increased expression of MYB2, and the transient expression of *DbMYB2* in *N. benthamiana* showed elevated expression of endogenous anthocyanin genes and thereby the increasing anthocyanin levels [166]. A transcriptomic study based on two *Paphiopedilum* species with green and tessellated leaves revealed that their differentially expressed genes were mostly enriched in processes like chloroplast, cytoplasm, thylakoid membrane, and nucleus [167]. For a *Pha. aphrodite* mutant with leaf variegation, the functional deficiency of PHOTOSYSTEM II SUBUNIT P (PsbP) protein, the extrinsic subunit of the photosystem II, is considered to play an important role in the formation of leaf variegation [168].

### Flowering time

Vegetative-to-reproductive transition is a key phase for orchid development and flowering regulation is critical in commercial orchid production. The floral transition of orchids is influenced by environmental cues such as ambient temperature and photoperiod [169], endogenous signals including phytohormones like gibberellic acid (GA) [170] and ABA [171], as well as genetic factors. The regulatory mechanism of orchid flowering varies in different species due to differing growth conditions while several flowering-related genes, either flowering promoters or repressors that have been functionally identified in *Phalaenopsis*, *Dendrobium*, and *Oncidium*, etc. show a similar pattern to affect flower transition [172–176]. It has been reported in *C. goeringii* and *Phalaenopsis* that ectopic overexpression of *FLOWERING LOCUS T* (*FT*) gene can lead to an earlier flowering phenotype in transgenic *Arabidopsis*, with significant upregulation of other flowering time-related genes [177, 178]. The floral meristem identity gene *LEAFY* accumulates high transcript levels in the floral meristem primordia of *Pha. aphrodite*, and its overexpression in rice results in precocious headings [174]. Also, the VIGS-*PhapLFY Pha. aphrodite* showed abnormal flower phenotypes like reduced pigmentation and morphological alterations in cell epidermis. Other orchid flowering promoters include *CONSTANS* (*CO*), *SUPPRESSOR OF OVEREXPRESSION OF CONSTANS 1* (*SOC1*), *APETALA1* (*AP1*) can also lead to early-flowering phenotypes in different types of transgenic plants examined [179–181]. While overexpression of *SHORT VEGETATIVE PHASE* (*SVP*) in *C. sinense* can significantly inhibit the expression of *FT*, *SOC1* and *APETALA1* (*AP1*) [102]. *Phalaenopsis SVP* also showed negative regulation in floral transition by repressing the expression of *PhaFT*s [182]. Another flowering repressor *TERMINAL FLOWER1* (*TFL1*) plays an antagonistic role with *FT* in repressing flowering, and the overexpression of *TFL* in *Dendrobium* causes delayed flowering and defective floral traits [175, 183].

Moreover, TFs such as MADS-box and zinc finger are thought to play an important role in mediating multiple signaling pathways in orchid flowering [184, 185]. miRNAs are also suggested to be involved in flowering regulation by targeting key genes that function in vegetative to reproductive transition [186–188]. In sum, the current understanding of the regulatory network of orchid flowering is based on the well-characterized genes from model plants, novel regulating factors of floral transition remain to be investigated. Also, the next challenge is to establish reliable protocols to make alterations in the flowering time of orchids to meet the market demand.

### Other properties

Genes related to other phenotypic traits or biological processes have been characterized in orchids. For example, secondary metabolites are critical in plant physiology in terms of stress and disease resistance. Functional verification of C-glycosyltransferase (CGT) genes was conducted in *D. catenatum*, which could specifically catalyze O-glycosylation (OGT) that helps drought resistance [189]. *Phalaenopsis* is distinguished by having long-lasting flowers; this feature is determined by the presence of cuticular waxes in perianth [190]. Ectopic overexpression of *R2R3-MYB* genes (*PaMYB9A1* and *PaMYB9A2*) of *Pha. aphrodite* in transgenic tobacco gives rise to a shiny leaf phenotype, indicating these two genes act in regulating the biosynthesis of cuticular wax [191]. WRKY and ARFs gene family have been functionally studied for *C. goeringii*, and their expression in transgenic *Arabidopsis* was highly upregulated after ABA and Indoleacetic acid (IAA) treatment, respectively, suggestive of their roles in response to abiotic stress [192, 193]. These functional genomics studies have elevated the possibilities to verify genes associated with numerous regulating networks and link them with desired traits, thus providing valuable resources for orchid molecular breeding and industrialization.

## Biotechnology-aided orchid production

Orchids exhibit several merits by having the elegant appearance, extended longevity, and varieties of cultivars that thrived in floriculture and gained widespread popularity among growers and consumers. Orchid has taken a significant position in the flower market, constituting more than 10% of the international pot plant trade with a 3.0% average annual growth in global import of cut flowers [7]. In addition to ornamental value, orchids such as *Dendrobium*, *Gastrodia*, and *Bletilla* have long been cultivated for medicinal use in Asia and Europe [194], and some *(Vanilla*) have been used for human consumption as flavorings and beverages [8]. Although remaining on a small scale, extracts of orchids have also been gradually applied and traded in the cosmetic, personal care products, and fragrance industries [195]. The significant increase in production and consumption of orchids is largely owing to the utilization of advanced breeding and cultivation techniques. Over the past two decades, tissue culture techniques associated with greenhouse cultivation has largely sped up orchid industrialization. More importantly, advanced biotechnology such as *Agrobacterium*-mediated transformation, particle bombardment and CRISPR/Cas9 genome editing for molecular breeding and large-scale propagation were more likely to be applied to the orchid industry for producing novel varieties with a longer shelf life and shortened production period that contribute to successful commercial orchid farming.

### Orchid propagation

Propagation techniques have gained major industrial importance for ornamental plants like orchids in which the commercial demand far exceeds the natural regeneration. Asymbiotic seed germination is a common way for orchid multiplication but it’s difficult for some terrestrial orchids (e.g. *Cypripedium*) and less favorable in large-scale production owing to the long juvenile period and heterozygosity in progenies [196]. To obtain propagated orchids with genetic stability and uniformity, micropropagation, usually by diverse forms of tissue culture (e.g. protocorm-like bodies (PLBs)) has been routinely used for mass commercial production and regeneration of endangered orchids [197]. Propagation protocols have been established for many species with commercial values and conservation purposes [198,199]. In spite of the advantages, somaclonal variations induced by prolonged clonal propagation and cryptic genetic effects under microenvironment become the major limitations for maintaining certain desired traits [200]. Therefore, molecular markers have been applied to examine the genetic variability of *in vitro* raised plants to ensure clonal fidelity [201, 202]. In this context, it has been pinpointed that biotechnological approaches will be a promising tool for orchid micropropagation and simultaneously, commercial production.

Conventionally, it takes three to 13 years to produce a flowering plant from seeds and the transition from the juvenile period to reproductive development is restricted by some environmental conditions such as vernalization [203]. Intriguingly, under a certain combination of growing conditions, orchid protocorms are able to bypass the vegetative phase and directly flower without leaf initiation and root development. It’s reported that this process is co-regulated by key TFs like KNOX, R2R3-MYB and Ovate Family Protein (OFP) that can induce a different flowering program [204]. This rapid *in vitro* flowering is of great potential in commercial utilization, especially for species with a long juvenile period. In addition to the reduction of reproduction cycle, enhancing the production of useful secondary metabolites has become an important objective for *in vitro* orchid propagation. Micropropagated orchids with treatment of illumination, abiotic and biotic stress, bioreactors or precursors showed an accumulation of secondary metabolites, indicative of their positive roles in end product production [205–207]. Overall, technical innovations have built the path from laboratory to commercialization for orchid micropropagation, which is expected to gain momentum and give rise to more elite orchid varieties and byproducts.

### Transgenic breeding

#### Genetic transformation

There are mainly two types of gene manipulation techniques, overexpression of exogenous genes (genetic transformation) and silencing of endogenous genes (gene editing/silencing). Genetic transformation is a sought-after technology for introducing agronomically useful genes to modify or recombine already existing traits into target plant species, expanding the gene pool beyond what has been available to conventional breeding means and does not exist in the species of interest. Also, traditional breeding strategies such as crossbreeding with the purpose of improving particular traits are time-consuming for orchids that have prolonged reproductive cycles and multiple backcrossing attempts are required. Therefore, genetic transformation poses a tremendous potential for efficient genetic enhancement of important ornamental plants like orchids. *Agrobacterium*-mediated approach and particle bombardment are two major strategies employed for transgenic orchids [208]. These two methods have been increasingly applied to studies with the aim of transferring desired genes to orchids that could give rise to commercially important traits, such as genes regulating novel flower color, flowering time, plant-pathogen/virus resistance, and cold tolerance (Table 3). These attempts provided efficient, possibly heritable and promising gene transformation to genetically modify specific characteristics of orchids. However, although bioinformatics analyses have provided implications for a wide spectrum of genes associated with orchid characteristics, only a small proportion of these have been functionally verified via gene transformation studies. Besides, it can take many years of painstaking research to develop transformation methods for different species as some orchids (e.g. *Cypripedium*) are recalcitrant for *in vitro* propagation, as mentioned earlier, plus the long reproduction cycle and the limited availability of genomic data. After the transgenic plant with the target gene is successfully generated, it is possible that the desired phenotype and the associated traits could be altered by the occurrence of somaclonal variation during propagation [208]. So far, transgenic studies have been done exclusively within the Epidendroideae subfamily, with *Cymbidium*, *Dendrobium*, *Oncidium*, *Vanda*, and *Phalaenopsis* as the representative genera for fundamental research and breeding applications. A routine protocol of genetic transformation for other taxa is still considerably lagging behind. Nevertheless, with the help of the ongoing release of high-quality whole genome sequencing in more orchids and the advent of cutting-edge techniques in molecular biology, our understanding of intrinsic mechanisms and modifications of genes of interest for desired traits of orchids will be fundamentally improved.

**Table 3:** Transgenic orchids with commercially applicable traits.

**Species/Cultivars**	**Explant**	**Methods**	**Target genes**	**Aims**	**References**
*Phalaenopsis* Taisuco Kochdiam × *Phalaenopsis* Taisuco Kaaladian	Protocorm-like bodies	Particle bombardment	*Cymbidium* Mosaic Virus (CymMV) coat protein cDNA	Improve the resistance of *Phalaenopsis* to CymMV	[225]
*Phalaenopsis* Amabilis W1–10 × *Phalaenopsis* Amabilis W1–22	Protocorm-like bodies	Particle bombardment and *Agr. tumefaciens* strain EHA105- mediated transformation	CymMV coat protein cDNA	Enable expression of viral and bacterial disease (CymMV) and *Erwinia carotovora* resistant traits	[226]
*Phalaenopsis amabilis* ‘Queen Beer, No.1227’	Protocorm-like bodies	*Agr. tumefaciens* strain LBA4404, vector: pCAMBIA2300	Lipid transfer protein (LTP) encoding gene	Enhance cold resistance in *Phalaenopsis*	[227]
*Phalaenopsis* Sogo Yukidian ‘V3’, *Phalaenopsis* OX Honey ‘OX1372’	Flower	Transient overexpression, *Agr. tumefaciens* strain EHA105- mediated transformation	*flavonoid-3′,5′-hydroxylase* (*F3’5’H*)	Assessment of *F3’5’H* ‘s function in causing violet-blue pigmentation in *Phalaenopsis* flowers	[228]
*Phalaenopsis* Sogo Yukidian ‘SPM313’	Protocorm-like bodies	*Agr. tumefaciens* strain EHA105- mediated transformation, vector: *Ubi:OsGA2ox6*	Rice GA2-oxidase gene *OsGA2ox6*	Detect GA2-oxidase gene’s role in miniaturizing *Phalaenopsis* species	[229]
*Phalaenopsis equestris*	Protocorm	*Agr. tumefaciens* mediated transformation, vector: pH2GW7	Orchid stress-associated protein (*SAP*) gene, *Pha21*	Elucidate the key role of *Pha21* in antiviral immunity enhancement of the RNAi pathway	[230]
*Erycina pusilla*	Protocorm	*Agr. tumefaciens* strains EHA105- mediated transformation, vector: p35S-AtMSRB7	*Methionine sulfoxide reductase B7* (*MSRB7*)	Verify *MSRB* gene’s function in response to abiotic stress in orchids	[231]
*Dendrobium* Madame Thong-In	Protocorm-like bodies	*Agr. tumefaciens* strain LBA4404 mediated transformation	*Dendrobium* Class I KNOX gene *DOH1*	Detect *DOH1*’s function in shaping plant architecture in *Dendrobium*	[232]
*Dendrobium* Sonia Earsakul	Protocorm-like bodies	Particle bombardment	CymMV coat protein cDNA	Generate resistance to CymMV in naturally CymMV-infected *Dendrobium*	[233]
*Dendrobium* Chao Praya Smile	Callus	Particle bombardment, vector: pGreen	Dendrobium *SOC1*	Verify *SOC1* could promote flowering in *Dendrobium*	[180]
*Dendrobium* Chao Praya Smile	Callus	*Agr. tumefaciens* -mediated transformation	*Dendrobium AP1*	Verify *AP1* could induce early flowering in *Dendrobium*	[173]
*Dendrobium* Chao Praya Smile	Callus	Particle bombardment *Agr. tumefaciens*-mediated transformation	*Dendrobium FT* and *FT*-*INTERACTING PROTEIN 1* (*FTIP1*)	Verify that *FT* interacts with *FTIP1* affecting flowering in *Dendrobium*	[234]
*Dendrobium* Sonia ‘Earsakul’	Protocorm-like bodies	*Agr. tumefaciens* -mediated transformation	*1-aminocyclopropane-1-carboxylic acid oxidase* (*ACO*)	Demonstrate the effect of reduced antisense ACO activity to vegetative and reproductive development	[235]
*Oncidium* ‘Sweet Sugar’ *Oncidium* ‘Sharry Baby’ *Odontoglossum* ‘Stirling Tiger’ *Odontoglossum* ‘Hansueli Isler’	Protocorm-like bodies	*Agr. tumefaciens* -mediated transformation	*Ethylene receptor mutant* (*etr1*)*-1*gene	Reduce flower’s sensitivity to exogenous ethylene and prolong display life	[236]
*Oncidium* Gower Ramsey	Protocorm-like bodies	*Agr. tumefaciens* - strain LBA4404 mediated transformation	*Oncidium* MADS box gene *OSMADS1*	Verify *OSMADS1* can promote early flowering of *Oncidium*	[237]

#### Genome editing

Genome editing refers to a group of newfangled genetic engineering technologies, in which programmed nucleases composed of sequence-specific DNA-binding domains are employed to induce targeted DNA double-strand breaks (DSBs) in the host that stimulate the cellular DNA repair mechanisms [209]. These site-specific nuclease technologies enable a specific target DNA to be added, removed, or altered at particular locations in the genome, providing great opportunities for plant genome engineering [210]. Before the emergence of sequence-specific nucleases, RNAi-mediated gene knock-down represented an efficient, low-cost, and high-throughput alternative to target gene silencing by homology-directed recombination [211]. There were several flower color/pigmentation-related studies conducted using gene knock-down by RNAi in orchids, including *Dendrobium* Sonia [212], *Oncidium* Gower Ramsey [213] and *Oncidium* Honey Angel [214], whereas this RNAi technology has several drawbacks regarding unpredictable off-target effects and temporary loss-of-function, which could hinder the practical application to a certain extent, due to limited linkage between phenotype and genotype [209]. The clustered regularly interspaced short palindromic repeat (CRISPR)/CRISPR-associated protein 9 (Cas9) system represents a newly developed, efficient tool for introducing site-specific DSBs [215], by which the target gene is completely knocked out and genomic change can be maintained and heritable to the progeny. Although the well-established transformation systems are far from complete in Orchidaceae, this technology has been successfully applied in both natural specimens and their cultivars. Kui *et al.* [216] reported a successful knockout of five target genes (*C3H*, *C4H*, *4CL*, *CCR*, and *IRX*) involved in the lignocellulose biosynthesis pathway in *D. officinale* by CRISPR/Cas9 gene editing. By examining mutation rates of the locus of each target gene, they claimed the established CRISPR/Cas9 system can work efficiently to edit endogenous genes in *D. officinale* and introduce mutations. Three MADS-box genes (*MADS44*, *MADS36*, and *MADS8*) were edited by CRISPR/Cas for producing single-guide RNAs (sgRNAs) and generating different combinations of mutants in *Pha. equestris* [217]. This study illustrated the possibility that several sgRNAs can be constructed into a library and transformed to create mutants with multiple phenotypes. However, whether the editing procedures reported by these two studies can actually produce knock-out phenotypes in orchids remains unknown. Yet only two subsequent studies have provided validated phenotypes correlated with the knockout of target genes using CRISPR/Cas9 technology. In *Pha. amabilis*, phytoene desaturase (*PDS3*) gene which encodes a rate-limiting enzyme in carotenoid synthesis has been edited by CRISPR/Cas9 system and the transformants showed an albino phenotype [218]. Li *et al.* [219] generated a kilobase-scale genomic deletion at the *DOTFL1* locus of *Dendrobium* Chao Praya Smile. The results showed *dotfl1* exhibited earlier occurrence of flowering, pseudobulb formation and termination of inflorescence apices, which are more explicit phenotypes related to floral transition than the previous reported *DOTFL1* knockdown lines by RNAi-mediated silencing [183]. Whereas all these practices have not yet been verified if these edited phenotypes can be retained to F2 and F3 -selfed and -crossed progeny, the gap between research experiment and industry application is still waiting to be filled in. Albeit started late and still in its infancy, these studies showed the great potential of CRISPR/Cas9 systems in efficient and accurate modification of orchid traits and this technique has huge potential to be applied in breeding new and improved orchid varieties without lengthy procedures and unexpected variables led by crossing breeding.

## Future prospects

Over the past two decades, the explosion of genomic sequencing technologies combined with recent advances in transgenic techniques have revolutionized the basic and applied biology in orchids. With the wealth of phylogenetic information available, more and more controversial taxonomy of orchid groups has been well solved, framing a reliable evolutionary relationship for this extraordinarily diverse family. Conservation genetics based on molecular markers enable the effective translation of underlying threats into practical conservation measures that are tailored for different orchid communities. The ongoing release of orchid whole genomes has elevated the possibilities of multi-omics studies in orchids in terms of genome mapping, gene expression, comparative genomics, and functional validation. The progress in these studies provides new insights into the origin, evolution, and diversification of Orchidaceae, as well as a molecular vision that uncovers the underlying regulating mechanisms pertaining to exquisite floral morphology, complex life histories, and unrivaled reproductive strategies. As genetic transformation and genome editing have become increasingly routine techniques in plant genome engineering, precise gene characterization and modification are feasible in orchids with complex genomes. Besides, tools like CRISPR-Cas9 system offers new opportunities for gene stacking, in which adding genes-of-interest with desired traits or knocking out genes associated with undesirable traits can be achieved simultaneously in a single practice [217, 220]. Although the current achievement of genome engineering is limited to orchids, these swift and robust approaches pave a way for efficient genetic improvement of important traits in orchids in the foreseeable future.

Apparently, we are now entering a post-genomic era for orchid research and industrialization. Despite the recent advances having unveiled many facets of this intriguing family, its scientific significance has not yet been fully realized. In addition, the deficiency of genome data in most orchid species still hampers the in-depth comparative genomics analysis. Genetic investigation and functional analysis are mainly focusing on well-characterized gene families such as MADS-box, MYB that have been extensively studied in model plants, with fewer efforts put into the inspection of novel regulating factors. Last but not least, genetic transformation protocols available at present are derived from several model orchids like *Dendrobium* and *Phalaenopsis*, which may not be applicable to many other orchid species with specified features, plus the existing transformation efficiency is relatively low in orchids compared to model plants such as *Arabidopsis* and rice; these pending problems will require multi-facet investments to guide future acts. Taken together, the ongoing accomplishments in developing novel genomic tools and techniques have shed new light onto orchid research and production. Our critical next step is to utilize their full potential in the application for orchid conservation, breeding, and industrialization.

## Acknowledgements

This work was supported by The National Key Research and Development Program of China (2019YFD1000400).

## Conflict of interests

The authors declare that they have no conflict of interests.

## References

[1] Chase MW , Cameron KM, Freudenstein JV et al. An updated classification of Orchidaceae. Bot J Linn Soc. 2015;177:151–74.

[2] Christenhusz MJM , Byng JW. The number of known plants species in the world and its annual increase. Phytotaxa. 2016;261:201–17.

[3] Cai J , Liu X, Vanneste K et al. The genome sequence of the orchid *Phalaenopsis equestris*. Nat Genet. 2015;47:65–72. 25420146 10.1038/ng.3149

[4] Phillips RD , Reiter N, Peakall R. Orchid conservation: from theory to practice. Ann Bot. 2020;126:345–62. 32407498 10.1093/aob/mcaa093 PMC7424752

[5] CITES . The CITES appendices 2021*.* https://cites.org/eng/app/appendices.php (31 March 2022, date last accessed).

[6] IUCN . The IUCN red list of threatened species 2021–3. https://www.iucnredlist.org/search?taxonomies=101295&searchType=species (31 March 2022, date last accessed).

[7] Yuan SC , Lekawatana S, Amore TD et al. The Global Orchid Market. In: Chen FC, Chin SW, eds. The Orchid Genome. Springer: Cham, 2021, 1–28.

[8] Hinsley A , de Boer HJ, Fay MF et al. A review of the trade in orchids and its implications for conservation. Bot J Linn Soc. 2018;186:435–55.

[9] Fay MF . Orchid conservation: how can we meet the challenges in the twenty-first century? Bot Stud. 2018;59:1–6. 29872972 10.1186/s40529-018-0232-z PMC5988927

[10] Chen C . CiteSpace II: detecting and visualizing emerging trends and transient patterns in scientific literature. J Am Soc Inf Sci Technol. 2006;57:359–77.

[11] Ahrens CW , Supple MA, Aitken NC et al. Genomic diversity guides conservation strategies among rare terrestrial orchid species when taxonomy remains uncertain. Ann Bot. 2017;119:1267–77. 28334284 10.1093/aob/mcx022 PMC5604565

[12] Cozzolino S , Nardella AM, Impagliazzo S et al. Hybridization and conservation of Mediterranean orchids: should we protect the orchid hybrids or the orchid hybrid zones? Biol Conserv. 2006;129:14–23.

[13] Agarwal M , Shrivastava N, Padh H. Advances in molecular marker techniques and their applications in plant sciences. Plant Cell Rep. 2008;27:617–31. 18246355 10.1007/s00299-008-0507-z

[14] Zhai JW , Zhang GQ, Chen LJ et al. A new orchid genus, *Danxiaorchis*, and phylogenetic analysis of the tribe Calypsoeae. PLoS One. 2013;8:e60371. 23593204 10.1371/journal.pone.0060371 PMC3617198

[15] Jin WT , Jin XH, Schuiteman A et al. Molecular systematics of subtribe Orchidinae and Asian taxa of Habenariinae (Orchideae, Orchidaceae) based on plastid *matK*, *rbcL* and nuclear *ITS*. Mol Phylogenet Evol. 2014;77:41–53. 24747003 10.1016/j.ympev.2014.04.004

[16] Jin W , Xiang X, Jin X et al. Generic delimitation of Orchidaceae from China: current situation and perspective. Biodivers Sci. 2015;23:237–42.

[17] Zhang GQ , Li MH, Su YY et al. A new myco-heterotrophic genus, *Yunorchis*, and the molecular phylogenetic relationships of the tribe Calypsoeae (Epidendroideae, Orchidaceae) inferred from plastid and nuclear DNA sequences. PLoS One. 2015;10:1–17. 10.1371/journal.pone.0123382 PMC4406536 25902264

[18] Xiang XG , Li DZ, Jin WT et al. Phylogenetic placement of the enigmatic orchid genera *Thaia* and *Tangtsinia*: evidence from molecular and morphological characters. Taxon. 2012;61:45–54.

[19] Liu DK , De TX, Zhao Z et al. Plastid phylogenomic data yield new and robust insights into the phylogeny of *Cleisostoma–Gastrochilus* clades (Orchidaceae, Aeridinae). Mol Phylogenet Evol. 2020;145:106729. 31926307 10.1016/j.ympev.2019.106729

[20] Huang WC , Liu ZJ, Jiang K et al. Phylogenetic analysis and character evolution of tribe Arethuseae (Orchidaceae) reveal a new genus *Mengzia*. Mol Phylogenet Evol. 2022;167:107362. 34775057 10.1016/j.ympev.2021.107362

[21] Li M , Yuan X, Liu D et al. *Bulbophyllum yunxiaoense* sp*.* nov. (Orchidaceae: Epidendroideae: Malaxideae) from Fujian, China: morphological and molecular analyses. Phytotaxa. 2017;332:59–66.

[22] Liao XY , Zhang DY, Lan SR et al. *Paphiopedilum erythroanthum*, a new species of slipper orchid (Cypripedioideae, Orchidaceae) from China based on morphological and molecular data. Phytotaxa. 2019;406:271–8.

[23] Ma L , Chen XY, Liu JF et al. *Gastrodia fujianensis* (Orchidaceae, Epidendroideae, Gastrodieae), a new species from China. Phytotaxa. 2019;391:269–77.

[24] Zhou Z , Zhang S, Yang YP et al. Morphological and molecular evidence for a new species from China: *Dendrobium yongjiaense* (Orchidaceae: Malaxideae). Phytotaxa. 2020;441:203–10.

[25] Tu X , Huang MZ, Liu DK et al. *Liparis mai* (Orchidaceae; Malaxideae), a new species from China: evidence from morphological and molecular analyses. Phytotaxa. 2021;435:235–42.

[26] Zhang D , Tu X, Liu B et al. *Cymbidium biflorens* (Orchidaceae; Epidendroideae), a new species from China: evidence from morphological and molecular data. Phytotaxa. 2020;428:271–8.

[27] Hu WQ , Zhang QH, Chen GZ et al. *Cymbidium motuoense* (Orchidaceae; Epidendroideae), a new species from China: evidence from morphological and molecular data. Phytotaxa. 2021;509:102–12.

[28] Davis CC , Xi Z, Mathews S. Plastid phylogenomics and green plant phylogeny: almost full circle but not quite there. BMC Biol. 2014;12:11. 24533863 10.1186/1741-7007-12-11 PMC3925952

[29] Chang CC , Lin HC, Lin IP et al. The chloroplast genome of *Phalaenopsis aphrodite* (Orchidaceae): comparative analysis of evolutionary rate with that of grasses and its phylogenetic implications. Mol Biol Evol. 2006;23:279–91. 16207935 10.1093/molbev/msj029

[30] Chase MW , Cameron KM, Barrett RL et al. DNA data and Orchidaceae systematics: a new phylogenetic classification. Orchid Conserv. 2003;69:32.

[31] Givnish TJ , Spalink D, Ames M et al. Orchid phylogenomics and multiple drivers of their extraordinary diversification. Proc R Soc B Biol Sci. 2015;282:20151553. 10.1098/rspb.2015.1553 PMC4571710 26311671

[32] Li YX , Li ZH, Schuiteman A et al. Phylogenomics of Orchidaceae based on plastid and mitochondrial genomes. Mol Phylogenet Evol. 2019;139:106540. 31252068 10.1016/j.ympev.2019.106540

[33] Serna-Sánchez MA , Pérez-Escobar OA, Bogarín D et al. Plastid phylogenomics resolves ambiguous relationships within the orchid family and provides a solid timeframe for biogeography and macroevolution. Sci Rep. 2021;11:1–11. 33767214 10.1038/s41598-021-83664-5 PMC7994851

[34] Tu XD , Liu DK, Xu SW et al. Plastid phylogenomics improves resolution of phylogenetic relationship in the *Cheirostylis* and *Goodyera* clades of Goodyerinae (Orchidoideae, Orchidaceae). Mol Phylogenet Evol. 2021;164:107269. 34324956 10.1016/j.ympev.2021.107269

[35] Niu Z , Xue Q, Wang H et al. Mutational biases and GC-biased gene conversion affect GC content in the plastomes of *Dendrobium* genus. Int J Mol Sci. 2017;18:2307. 29099062 10.3390/ijms18112307 PMC5713276

[36] Yang JB , Tang M, Li HT et al. Complete chloroplast genome of the genus *Cymbidium*: lights into the species identification, phylogenetic implications and population genetic analyses. BMC Evol Biol. 2013;13:1–12. 23597078 10.1186/1471-2148-13-84 PMC3644226

[37] Li ZH , Ma X, Wang DY et al. Evolution of plastid genomes of *Holcoglossum* (Orchidaceae) with recent radiation. BMC Evol Biol. 2019;19:1–10. 30808310 10.1186/s12862-019-1384-5 PMC6390633

[38] Guo YY , Yang JX, Bai MZ et al. The chloroplast genome evolution of Venus slipper (*Paphiopedilum*): IR expansion, SSC contraction, and highly rearranged SSC regions. BMC Plant Biol. 2021;21:1–14. 34058997 10.1186/s12870-021-03053-y PMC8165784

[39] Deng H , Zhang GQ, Lin M et al. Mining from transcriptomes: 315 single-copy orthologous genes concatenated for the phylogenetic analyses of Orchidaceae. Ecol Evol. 2015;5:3800–7. 26380706 10.1002/ece3.1642 PMC4567881

[40] Guo YY , Zhang YQ, Zhang GQ et al. Comparative transcriptomics provides insight into the molecular basis of species diversification of section *Trigonopedia* (*Cypripedium*) on the Qinghai-Tibetan plateau. Sci Rep. 2018;8:11640. 30076357 10.1038/s41598-018-30147-9 PMC6076244

[41] Unruh SA , McKain MR, Lee YI et al. Phylotranscriptomic analysis and genome evolution of the Cypripedioideae (Orchidaceae). Am J Bot. 2018;105:631–40. 29608785 10.1002/ajb2.1047

[42] Piñeiro Fernández L , Byers K, Cai J et al. A phylogenomic analysis of the floral transcriptomes of sexually deceptive and rewarding European orchids, *Ophrys* and *Gymnadenia*. Front Plant Sci. 2019;10:1553. 31850034 10.3389/fpls.2019.01553 PMC6895147

[43] Wong DCJ , Peakall R. Orchid phylotranscriptomics: the prospects of repurposing multi-tissue transcriptomes for phylogenetic analysis and beyond. Front Plant Sci. 2022;13:910362. 35712597 10.3389/fpls.2022.910362 PMC9196242

[44] Gale SW , Fischer GA, Cribb PJ et al. Orchid conservation: bridging the gap between science and practice. Bot J Linn Soc. 2018;186:425–34.

[45] Chung MY , Nason JD, Chung MG. Spatial genetic structure in populations of the terrestrial orchid *Cephalanthera longibracteata* (Orchidaceae). Am J Bot. 2004;91:52–7. 21653362 10.3732/ajb.91.1.52

[46] Forrest A , Hollingsworth M, Hollingsworth P et al. Population genetic structure in European populations of *Spiranthes romanzoffiana* set in the context of other genetic studies on orchids. Heredity. 2004;92:218–27. 14666133 10.1038/sj.hdy.6800399

[47] Yang Q , Fu Y, Wang Y et al. Genetic diversity and differentiation in the critically endangered orchid (*Amitostigma hemipilioides*): implications for conservation. Plant Syst Evol. 2014;300:871–9.

[48] Chung MY , Park CW. Fixation of alleles and depleted levels of genetic variation within populations of the endangered lithophytic orchid *Amitostigma gracile* (Orchidaceae) in South Korea: implications for conservation. Plant Syst Evol. 2008;272:119–30.

[49] Tian HZ , Han LX, Zhang JL et al. Genetic diversity in the endangered terrestrial orchid *Cypripedium japonicum* in East Asia: insights into population history and implications for conservation. Sci Rep. 2018;8:6467. 29691494 10.1038/s41598-018-24912-z PMC5915404

[50] Guo JL , Cao WJ, Li ZM et al. Conservation implications of population genetic structure in a threatened orchid *Cypripedium tibeticum*. Plant Divers. 2019;41:13–8. 30931413 10.1016/j.pld.2018.12.002 PMC6412106

[51] Brzosko E , Wróblewska A, Tałałaj I et al. Genetic diversity of *Cypripedium calceolus* in Poland. Plant Syst Evol. 2011;295:83–96.

[52] Pedersen HÆ , Rasmussen HM, Kahandawala IM et al. Genetic diversity, compatibility patterns and seed quality in isolated populations of *Cypripedium calceolus* (Orchidaceae). Conserv Genet. 2012;13:89–98.

[53] Fay MF , Bone R, Cook P et al. Genetic diversity in *Cypripedium calceolus* (Orchidaceae) with a focus on northwestern Europe, as revealed by plastid DNA length polymorphisms. Ann Bot. 2009;104:517–25. 19454594 10.1093/aob/mcp116 PMC2720647

[54] Gargiulo R , Ilves A, Kaart T et al. High genetic diversity in a threatened clonal species, *Cypripedium calceolus* (Orchidaceae), enables long-term stability of the species in different biogeographical regions in Estonia. Bot J Linn. Soc. 2018;186:560–71.

[55] Hietz P , Buchberger G, Winkler M. Effect of forest disturbance on abundance and distribution of epiphytic bromeliads and orchids. Ecotropica. 2006;12:103–12.

[56] Jaros U , Fischer G, Pailler T et al. Spatial patterns of AFLP diversity in *Bulbophyllum occultum* (Orchidaceae) indicate long-term refugial isolation in Madagascar and long-distance colonization effects in La Réunion. Heredity. 2016;116:434–46. 26883184 10.1038/hdy.2016.1 PMC4834385

[57] Hu AQ , Gale SW, Kumar P et al. Preponderance of clonality triggers loss of sex in *Bulbophyllum bicolor*, an obligately outcrossing epiphytic orchid. Mol Ecol. 2017;26:3358–72. 28390097 10.1111/mec.14139

[58] Cozzolino S , Widmer A. Orchid diversity: An evolutionary consequence of deception? Trends Ecol Evol. 2005;20:487–94. 16701425 10.1016/j.tree.2005.06.004

[59] Fajardo CG , de Almeida VF, Felix LP et al. Negligence in the Atlantic forest, northern Brazil: a case study of an endangered orchid. Biodivers Conserv. 2017;26:1047–63.

[60] Gomes PCL , de Camargo SE, de Fraga CN et al. High genetic variability is preserved in relict populations of *Cattleya lobata* (Orchidaceae) in the Atlantic rainforests inselbergs. Rev Bras Bot. 2018;41:185–95.

[61] Blambert L , Mallet B, Humeau L et al. Reproductive patterns, genetic diversity and inbreeding depression in two closely related *Jumellea* species with contrasting patterns of commonness and distribution. Ann Bot. 2016;118:93–103. 26944785 10.1093/aob/mcw014 PMC4934390

[62] Ross AA , Travers SE. The genetic consequences of rarity in the western prairie fringed orchid (*Platanthera praeclara*). Conserv Genet. 2016;17:69–76.

[63] John ALDW , Mäder G, Fregonezi JN et al. Genetic diversity and population structure of naturally rare *Calibrachoa* species with small distribution in southern Brazil. Genet Mol Biol. 2019;42:108–19. 30856243 10.1590/1678-4685-GMB-2017-0314 PMC6428134

[64] Wagner ND , Clements MA, Simpson L et al. Conservation in the face of hybridisation: genome-wide study to evaluate taxonomic delimitation and conservation status of a threatened orchid species. Conserv Genet. 2021;22:151–68.

[65] Jersáková J , Johnson S, Kindlmann P. Mechanisms and evolution of deceptive pollination in orchids. Biol Rev. 2006;81:219–35. 16677433 10.1017/S1464793105006986

[66] Schlüter PM , Schiestl FP. Molecular mechanisms of floral mimicry in orchids. Trends Plant Sci. 2008;13:228–35. 18424223 10.1016/j.tplants.2008.02.008

[67] Cardoso-Gustavson P , Saka MN, Pessoa EM et al. Unidirectional transitions in nectar gain and loss suggest food deception is a stable evolutionary strategy in *Epidendrum* (Orchidaceae): insights from anatomical and molecular evidence. BMC Plant Biol. 2018;18:179. 30180799 10.1186/s12870-018-1398-y PMC6122447

[68] Peakall R , Ebert D, Poldy J et al. Pollinator specificity, floral odour chemistry and the phylogeny of Australian sexually deceptive *Chiloglottis* orchids: implications for pollinator-driven speciation. New Phytol. 2010;188:437–50. 20561345 10.1111/j.1469-8137.2010.03308.x

[69] Vereecken NJ , Wilson CA, Hötling S et al. Pre-adaptations and the evolution of pollination by sexual deception: Cope's rule of specialization revisited. Proc Biol Sci. 2012;279:4786–94. 23055065 10.1098/rspb.2012.1804 PMC3497092

[70] Bohman B , Flematti GR, Barrow RA et al. Pollination by sexual deception—it takes chemistry to work. Curr Opin Plant Biol. 2016;32:37–46. 27368084 10.1016/j.pbi.2016.06.004

[71] McCormick MK , Whigham DF, Canchani-Viruet A. Mycorrhizal fungi affect orchid distribution and population dynamics. New Phytol. 2018;219:1207e1215. 29790578 10.1111/nph.15223

[72] Li T , Wu S, Yang W et al. How mycorrhizal associations influence orchid distribution and population dynamics. Front Plant Sci. 2021;12:647114. 34025695 10.3389/fpls.2021.647114 PMC8138319

[73] Ma XY , Kang JC, Nontachaiyapoom S et al. Non-mycorrhizal endophytic fungi from orchids. Curr Sci 2015;109:72–87.

[74] Selosse MA , Petrolli R, Mujica MI et al. The waiting room hypothesis revisited by orchids: were orchid mycorrhizal fungi recruited among root endophytes? Ann Bot. 2022;129:259–70. 34718377 10.1093/aob/mcab134 PMC8835631

[75] Pecoraro L , Caruso T, Cai L et al. Fungal networks and orchid distribution: new insights from above- and below-ground analyses of fungal communities. IMA Fungus. 2018;9:1347. 10.5598/imafungus.2018.09.01.01 PMC6048571 30018868

[76] Suetsugu K , Yamato M, Matsubayashi J et al. Partial and full mycoheterotrophy in green and albino phenotypes of the slipper orchid *Cypripedium debile*. Mycorrhiza. 2021;31:301–12. 33852063 10.1007/s00572-021-01032-7

[77] Li MH , Liu KW, Li Z et al. Genomes of leafy and leafless *Platanthera* orchids illuminate the evolution of mycoheterotrophy. Nat Plants. 2022;8:373–88. 35449401 10.1038/s41477-022-01127-9 PMC9023349

[78] Dearnaley JD , Cameron DD. Nitrogen transport in the orchid mycorrhizal symbiosis-further evidence for a mutualistic association. New Phytol. 2016;213:10–2. 10.1111/nph.14357 27891646

[79] Kohler A , Kuo A, Nagy LG et al. Convergent losses of decay mechanisms and rapid turnover of symbiosis genes in mycorrhizal mutualists. Nat Genet. 2015;47:410–5. 25706625 10.1038/ng.3223

[80] Yang X , Cushman JC, Borland AM et al. A roadmap for research on crassulacean acid metabolism (CAM) to enhance sustainable food and bioenergy production in a hotter, drier world. New Phytol. 2015;207:491–504. 26153373 10.1111/nph.13393

[81] Deng H , Zhang LS, Zhang GQ et al. Evolutionary history of PEPC genes in green plants: implications for the evolution of CAM in orchids. Mol Phylogenet Evol. 2016;94:559–64. 26493226 10.1016/j.ympev.2015.10.007

[82] Zhang L , Chen F, Zhang GQ et al. Origin and mechanism of crassulacean acid metabolism in orchids as implied by comparative transcriptomics and genomics of the carbon fixation pathway. Plant J. 2016;86:175–85. 26959080 10.1111/tpj.13159

[83] Zhang Y , Dong W, Zhao X et al. Transcriptomic analysis of differentially expressed genes and alternative splicing events associated with crassulacean acid metabolism in orchids. Hortic Plant J. 2019;5:268–80.

[84] Bone RE , Smith JAC, Arrigo N et al. A macro‐ecological perspective on crassulacean acid metabolism (CAM) photosynthesis evolution in afro-Madagascan drylands: Eulophiinae orchids as a case study. New Phytol. 2015;208:469–81. 26192467 10.1111/nph.13572

[85] Ceusters N , Luca S, Feil R et al. Hierarchical clustering reveals unique features in the diel dynamics of metabolites in the CAM orchid *Phalaenopsis*. J Exp Bot. 2019;70:3269–81. 30972416 10.1093/jxb/erz170 PMC6598073

[86] Tay S , He J, Yam TW et al. CAM plasticity in epiphytic tropical orchid species responding to environmental stress. Bot Stud. 2019;60:1–15. 31087187 10.1186/s40529-019-0255-0 PMC6513927

[87] Sim SB , Corpuz RL, Simmonds TJ et al. HiFiAdapterFilt, a memory efficient read processing pipeline, prevents occurrence of adapter sequence in PacBio HiFi reads and their negative impacts on genome assembly. BMC Genomics. 2022;23:3196. 10.1186/s12864-022-08375-1 PMC8864876 35193521

[88] Koren S , Schatz MC, Walenz BP et al. Hybrid error correction and de novo assembly of single-molecule sequencing reads. Nat Biotechnol. 2012;30:693–700. 22750884 10.1038/nbt.2280 PMC3707490

[89] Jain M , Olsen HE, Paten B et al. The Oxford Nanopore MinION: delivery of nanopore sequencing to the genomics community. Genome Biol. 2016;17:239. 27887629 10.1186/s13059-016-1103-0 PMC5124260

[90] Chao YT , Chen WC, Chen CY et al. Chromosome-level assembly, genetic and physical mapping of *Phalaenopsis aphrodite* genome provides new insights into species adaptation and resources for orchid breeding. Plant Biotechnol J. 2018;16:2027–41. 29704444 10.1111/pbi.12936 PMC6230949

[91] Zhang GQ , Liu KW, Li Z et al. The *Apostasia* genome and the evolution of orchids. Nature. 2017;549:379–83. 28902843 10.1038/nature23897 PMC7416622

[92] Rasmussen HN . Terrestrial Orchids. In: From Seed to Mycotrophic Plant. Cambridge University Press: Cambridge, 1995.

[93] Jiang Y , Hu X, Yuan Y et al. The *Gastrodia menghaiensis* (Orchidaceae) genome provides new insights of orchid mycorrhizal interactions. BMC Plant Biol. 2022;22:179. 35392808 10.1186/s12870-022-03573-1 PMC8988336

[94] Yuan Y , Jin X, Liu J et al. The *Gastrodia elata* genome provides insights into plant adaptation to heterotrophy. Nat Commun. 2018;9:1615. 29691383 10.1038/s41467-018-03423-5 PMC5915607

[95] Xu Y , Lei Y, Su Z et al. A chromosome-scale *Gastrodia elata* genome and large-scale comparative genomic analysis indicate convergent evolution by gene loss in mycoheterotrophic and parasitic plants. Plant J. 2021;108:1609–23. 34647389 10.1111/tpj.15528

[96] Zhang GQ , Xu Q, Bian C et al. The *Dendrobium catenatum* Lindl. genome sequence provides insights into polysaccharide synthase, floral development and adaptive evolution. Sci Rep. 2016;6:19029. 26754549 10.1038/srep19029 PMC4709516

[97] Niu Z , Zhu F, Fan Y et al. The chromosome-level reference genome assembly for *Dendrobium officinale* and its utility of functional genomics research and molecular breeding study. Acta Pharm Sin B. 2021;11:2080–92. 34386340 10.1016/j.apsb.2021.01.019 PMC8343110

[98] Zhang Y , Zhang GQ, Zhang D et al. Chromosome-scale assembly of the *Dendrobium chrysotoxum* genome enhances the understanding of orchid evolution. Hortic Res. 2021;8:183. 34465765 10.1038/s41438-021-00621-z PMC8408244

[99] Jiang L , Lin M, Wang H et al. Haplotype-resolved genome assembly of *Bletilla striata* (Thunb.) Reichb. f. to elucidate medicinal value. Plant J. 2022;111:1340–53. 35785503 10.1111/tpj.15892

[100] Hasing T , Tang H, Brym M et al. A phased *Vanilla planifolia* genome enables genetic improvement of flavour and production. Nat Food. 2020;1:811–9. 10.1038/s43016-020-00197-2 37128067

[101] Piet Q , Droc G, Marande W et al. A chromosome-level, haplotype-phased *Vanilla planifolia* genome highlights the challenge of partial endoreplication for accurate whole-genome assembly. Plant Commun. 2022;3:100330. 35617961 10.1016/j.xplc.2022.100330 PMC9482989

[102] Yang FX , Gao J, Wei YL et al. The genome of *Cymbidium sinense* revealed the evolution of orchid traits. Plant Biotechnol J. 2021;19:2501–16. 34342129 10.1111/pbi.13676 PMC8633513

[103] Sun Y , Chen GZ, Huang J et al. The *Cymbidium goeringii* genome provides insight into organ development and adaptive evolution in orchids. Ornam Plant Res. 2021;1:10.

[104] Ai Y , Li Z, Sun WH et al. The *Cymbidium* genome reveals the evolution of unique morphological traits. Hortic Res. 2021;8:264. 34907207 10.1038/s41438-021-00709-6 PMC8671566

[105] Hsiao YY , Fu CH, Ho SY et al. OrchidBase 4.0: a database for orchid genomics and molecular biology. BMC Plant Biol. 2021;21:1–11. 34384382 10.1186/s12870-021-03140-0 PMC8359044

[106] Chao YT , Yen SH, Yeh JH et al. Orchidstra 2.0—a transcriptomics resource for the orchid family. Plant Cell Physiol. 2017;58:pcw220–e9. 10.1093/pcp/pcw220 28111366

[107] Lee CY , Viswanath KK, Huang JZ et al. Research article PhaLDB:a comprehensive database for molecular mining of the *Phalaenopsis* genome, transcriptome and miRNome. Genet Mol Res. 2018;17:18051.

[108] He C , Teixeira da Silva JA, Tan J et al. A genome-wide identification of the WRKY family genes and a survey of potential WRKY target genes in *Dendrobium officinale*. Sci Rep. 2017;7:9200. 28835632 10.1038/s41598-017-07872-8 PMC5569039

[109] Wang J , Liu Z, Zhang G et al. Evolution of two ubiquitin-like system of autophagy in orchid. Hortic Plant J. 2020;6:321–34.

[110] Chen YY , Hsiao YY, Chang S et al. Genome-wide identification of *YABBY* genes in Orchidaceae and their expression patterns in *Phalaenopsis* orchid. Genes. 2020;11:955. 32825004 10.3390/genes11090955 PMC7563141

[111] Fan H , Cui M, Li N et al. Genome-wide identification and expression analyses of R2R3-MYB transcription factor genes from two orchid species. PeerJ. 2020;8:e9781. 32953268 10.7717/peerj.9781 PMC7473048

[112] Wang QQ , Zhu MJ, Yu X et al. Genome-wide identification and expression analysis of terpene synthase genes in *Cymbidium faberi*. Front Plant Sci. 2021;12:751853. 34899778 10.3389/fpls.2021.751853 PMC8656225

[113] Mi ZY , Zhao Q, Lu C et al. Genome-wide analysis and the expression pattern of the MADS-box gene family in *Bletilla striata*. Plan Theory. 2021;10:2184. 10.3390/plants10102184 PMC8539064 34685993

[114] Ke YJ , Zheng QD, Yao YH et al. Genome-wide identification of the MYB gene family in *Cymbidium ensifolium* and its expression analysis in different flower colors. Int J Mol Sci. 2021;22:13245. 34948043 10.3390/ijms222413245 PMC8706735

[115] Zhang D , Lan S, Yin W et al. Genome wide identification and expression pattern analysis of KNOX gene family in Orchidaceae. Front Plant Sci. 2022;13:901089. 35712569 10.3389/fpls.2022.901089 PMC9197187

[116] Sedeek KEM , Scopece G, Staedler YM et al. Genic rather than genome-wide differences between sexually deceptive *Ophrys* orchids with different pollinators. Mol Ecol. 2014;23:6192–205. 25370335 10.1111/mec.12992

[117] Hsu CC , Chen SY, Chiu SY et al. High-density genetic map and genome-wide association studies of aesthetic traits in *Phalaenopsis* orchids. Sci Rep. 2022;12:1–15. 35228611 10.1038/s41598-022-07318-w PMC8885740

[118] Theißen G . Development of floral organ identity: stories from the MADS house. Curr Opin Plant Biol. 2001;4:75–85. 11163172 10.1016/s1369-5266(00)00139-4

[119] Mondragón-Palomino M , Theißen G. MADS about the evolution of orchid flowers. Trends Plant Sci. 2008;13:51–9. 18262819 10.1016/j.tplants.2007.11.007

[120] Pan ZJ , Cheng CC, Tsai WC et al. The duplicated B-class MADS-box genes display dualistic characters in orchid floral organ identity and growth. Plant Cell Physiol. 2011;52:1515–31. 21757456 10.1093/pcp/pcr092

[121] Hsu HF , Hsu WH, Lee YI et al. Model for perianth formation in orchids. Nat Plants. 2015;1:15046.

[122] Li X , Luo J, Yan T et al. Deep sequencing-based analysis of the *Cymbidium ensifolium* floral transcriptome. PLoS One. 2013;8:e85480. 24392013 10.1371/journal.pone.0085480 PMC3877369

[123] Sun Y , Wang G, Li Y et al. *De novo* transcriptome sequencing and comparative analysis to discover genes related to floral development in *Cymbidium faberi* Rolfe. Springerplus. 2016;5:1458. 27833829 10.1186/s40064-016-3089-1 PMC5082062

[124] Yang F , Zhu G, Wang Z et al. Integrated mRNA and microRNA transcriptome variations in the multi-tepal mutant provide insights into the floral patterning of the orchid *Cymbidium goeringii*. BMC Genomics. 2017;18:1–24. 28490318 10.1186/s12864-017-3756-9 PMC5426072

[125] Su S , Shao X, Zhu C et al. Transcriptome-wide analysis reveals the origin of peloria in Chinese *Cymbidium* (*Cymbidium sinense*). Plant Cell Physiol. 2018;59:2064–74. 29986119 10.1093/pcp/pcy130

[126] Pan ZJ , Chen YY, Du JS et al. Flower development of *Phalaenopsis* orchid involves functionally divergent *SEPALLATA*-like genes. New Phytol. 2014;202:1024–42. 24571782 10.1111/nph.12723 PMC4288972

[127] Shen CY , Chen YY, Liu KW et al. Orchid Bsister gene *PeMADS28* displays conserved function in ovule integument development. Sci Rep. 2021;11:1205. 33441740 10.1038/s41598-020-79877-9 PMC7806631

[128] Wang Y , Li Y, Yan X et al. Characterization of C- and D-class MADS-box genes in orchids. Plant Physiol. 2020;184:1469–81. 32900977 10.1104/pp.20.00487 PMC7608164

[129] Chen YY , Hsiao YY, Li CI et al. The ancestral duplicated DL/CRC orthologs, *PeDL1* and *PeDL2*, function in orchid reproductive organ innovation. J Exp Bot. 2021;72:5442–61. 33963755 10.1093/jxb/erab195

[130] Mitoma M , Kanno A. The greenish flower phenotype of *Habenaria radiata* (Orchidaceae) is caused by a mutation in the *SEPALLATA-like* MADS-box gene *HrSEP-1*. Front Plant Sci. 2018;9:831. 29971084 10.3389/fpls.2018.00831 PMC6018480

[131] Xu Z , Liu Q, Chen Y et al. miR390 family of *Cymbidium goeringii* is involved in the development of reproductive organs in transgenic Arabidopsis. BMC Plant Biol. 2022;22:149. 35346036 10.1186/s12870-022-03539-3 PMC8962573

[132] Valoroso MC , De Paolo S, Iazzetti G et al. Transcriptome-wide identification and expression analysis of *DIVARICATA*- and *RADIALIS*-like genes of the mediterranean orchid *Orchis italica*. Genome Biol Evol. 2017;9:1418–31. 10.1093/gbe/evx101 PMC5499889 28541415

[133] Tsai WC , Hsaio YY, Pan ZJ et al. The role of ethylene in orchid ovule development. Plant Sci. 2008;175:98–105.

[134] Chen Y , Xu Z, Shen Q et al. Floral organ-specific proteome profiling of the floral ornamental orchid (*Cymbidium goeringii*) reveals candidate proteins related to floral organ development. Bot Stud. 2021;62:23. 10.1186/s40529-021-00330-9 PMC8684572 34921643

[135] Rodrigues JA , Espley RV, Allan AC. Genomic analysis uncovers functional variation in the C-terminus of anthocyanin-activating MYB transcription factors. Hortic Res. 2021;8:77. 33790254 10.1038/s41438-021-00514-1 PMC8012628

[136] Hsu CC , Chen YY, Tsai WC et al. Three R2R3-MYB transcription factors regulate distinct floral pigmentation patterning in *Phalaenopsis* spp. Plant Physiol. 2015;168:175–91. 25739699 10.1104/pp.114.254599 PMC4424010

[137] Wang R , Mao C, Ming F. PeMYB4L interacts with PeMYC4 to regulate anthocyanin biosynthesis in *Phalaenopsis* orchid. Plant Sci. 2022;324:111423. 35995112 10.1016/j.plantsci.2022.111423

[138] Li BJ , Zheng BQ, Wang JY et al. New insight into the molecular mechanism of colour differentiation among floral segments in orchids. Commun Biol. 2020;3:89. 10.1038/s42003-020-0821-8 PMC7048853 32111943

[139] Hsu CC , Su CJ, Jeng MF et al. A HORT1 Retrotransposon insertion in the *PeMYB11* Promoter causes harlequin/black flowers in *Phalaenopsis* orchids. Plant Physiol. 2019;180:1535–48. 31088902 10.1104/pp.19.00205 PMC6752922

[140] Ramsay NA , Glover BJ. MYB–bHLH–WD40 protein complex and the evolution of cellular diversity. Trends Plant Sci. 2005;10:63–70. 15708343 10.1016/j.tplants.2004.12.011

[141] Zhang Y , Zhou T, Dai Z et al. Comparative transcriptomics provides insight into floral color polymorphism in a *Pleione limprichtii* orchid population. Int J Mol Sci. 2020;21:247. 10.3390/ijms21010247 PMC6982098 31905846

[142] Wong DC , Perkins J, Peakall R. Anthocyanin and flavonol glycoside metabolic pathways underpin floral color mimicry and contrast in a sexually deceptive orchid. Front Plant Sci. 2022;13:860997. 35401591 10.3389/fpls.2022.860997 PMC8983864

[143] Zhou Z , Ying Z, Wu Z et al. Anthocyanin genes involved in the flower coloration mechanisms of *Cymbidium kanran*. Front Plant Sci. 2021;12:737815. 34712257 10.3389/fpls.2021.737815 PMC8545884

[144] Li X , Fan J, Luo S et al. Comparative transcriptome analysis identified important genes and regulatory pathways for flower color variation in *Paphiopedilum hirsutissimum*. BMC Plant Biol. 2021;21:495. 10.1186/s12870-021-03256-3 PMC8549352 34706650

[145] Jeong SW , Das PK, Jeoung SC et al. Ethylene suppression of sugar-induced anthocyanin pigmentation in Arabidopsis. Plant Physiol. 2010;154:1514–31. 20876338 10.1104/pp.110.161869 PMC2971625

[146] Das PK , Shin DH, Choi SB et al. Cytokinins enhance sugar-induced anthocyanin biosynthesis in Arabidopsis. Mol Cells. 2012;34:93–101. 22699753 10.1007/s10059-012-0114-2 PMC3887782

[147] Li G , Zhao J, Qin B et al. ABA mediates development-dependent anthocyanin biosynthesis and fruit coloration in *Lycium* plants. BMC Plant Biol. 2019;19:317. 31307384 10.1186/s12870-019-1931-7 PMC6631627

[148] He C , Liu X, Teixeira da Silva JA et al. Transcriptome sequencing and metabolite profiling analyses provide comprehensive insight into molecular mechanisms of flower development in *Dendrobium officinale* (Orchidaceae). Plant Mol Biol. 2020;104:529–48. 32876816 10.1007/s11103-020-01058-z

[149] Cui X , Deng J, Huang C et al. Transcriptomic analysis of the anthocyanin biosynthetic pathway reveals the molecular mechanism associated with purple color formation in *Dendrobium Nestor*. Life. 2021;11:113. 33540822 10.3390/life11020113 PMC7912934

[150] Salzmann CC , Cozzolino S, Schiestl FP. Floral scent in food-deceptive orchids: species specificity and sources of variability. Plant Biol. 2007;9:720–9. 17891704 10.1055/s-2007-965614

[151] Vereecken NJ , Cozzolino S, Schiestl FP. Hybrid floral scent novelty drives pollinator shift in sexually deceptive orchids. BMC Evol Biol. 2010;10:103–12. 20409296 10.1186/1471-2148-10-103 PMC2875231

[152] Ramya M , Jang S, An HR et al. Volatile organic compounds from orchids: from synthesis and function to gene regulation. Int J Mol Sci. 2020;21:1160. 32050562 10.3390/ijms21031160 PMC7037033

[153] Ramya M , Park PH, Chuang YC et al. RNA sequencing analysis of *Cymbidium goeringii* identifies floral scent biosynthesis related genes. BMC Plant Biol. 2019;19:337. 10.1186/s12870-019-1940-6 PMC6679452 31375064

[154] Xu Q , Wang S, Hong H et al. Transcriptomic profiling of the flower scent biosynthesis pathway of *Cymbidium faberi* Rolfe and functional characterization of its jasmonic acid carboxyl methyltransferase gene. BMC Genomics. 2019;20:1–14. 30744548 10.1186/s12864-019-5501-z PMC6371524

[155] Huang M , Ma C, Yu R et al. Concurrent changes in methyl jasmonate emission and the expression of its biosynthesis-related genes in *Cymbidium ensifolium* flowers. Physiol Plant. 2015;153:503–12. 25214235 10.1111/ppl.12275

[156] Yeh CW , Zhong HQ, Ho YF et al. The diurnal emission of floral scent in oncidium hybrid orchid is controlled by *CIRCADIAN CLOCK ASSOCIATED 1 (CCA1)* through the direct regulation on terpene synthase. BMC Plant Biol. 2022;22:472. 36195835 10.1186/s12870-022-03850-z PMC9531428

[157] Chuang YC , Lee MC, Chang YL et al. Diurnal regulation of the floral scent emission by light and circadian rhythm in the *Phalaenopsis* orchids. Bot Stud. 2017;58:50. 29143225 10.1186/s40529-017-0204-8 PMC5688052

[158] Zhao C , Yu Z, Teixeira da Silva JA et al. Functional characterization of a *Dendrobium officinale* geraniol synthase doges1 involved in floral scent formation. Int J Mol Sci. 2020;21:7005. 10.3390/ijms21197005 PMC7582308 32977586

[159] Chuang YC , Hung YC, Tsai WC et al. PbbHLH4 regulates floral monoterpene biosynthesis in *Phalaenopsis* orchids. J Exp Bot. 2018;69:4363–77. 29982590 10.1093/jxb/ery246 PMC6093345

[160] Huang H , Kuo YW, Chuang YC et al. *Terpene synthase-b* and *terpene synthase-e/f* genes produce monoterpenes for *Phalaenopsis bellina* floral scent. Front Plant Sci. 2021;12:700958. 34335666 10.3389/fpls.2021.700958 PMC8318001

[161] Zhao MH , Li X, Zhang XX et al. Mutation mechanism of leaf color in plants: a review. Forests. 2020;11:851.

[162] Kim SH , Kim SW, Lim GH et al. Transcriptome analysis to identify candidate genes associated with the yellow-leaf phenotype of a *Cymbidium* mutant generated by γ-irradiation. PLoS One. 2020;15:e0228078. 10.1371/journal.pone.0228078 PMC6988911 31995594

[163] Yu J , Qiang W, Qin-Qin S et al. Transcriptome analysis reveals genes associated with leaf color mutants in *Cymbidium longibracteatum*. Tree Genet Genomes. 2020;16:10.1007/s11295-020-01440-4.

[164] Zhu G , Yang F, Shi S et al. Transcriptome characterization of *Cymbidium sinense* ‘dharma’ using 454 pyrosequencing and its application in the identification of genes associated with leaf color variation. PLoS One. 2015;10:e0128592. 26042676 10.1371/journal.pone.0128592 PMC4456352

[165] Gao J , Ren R, Wei Y et al. Comparative metabolomic analysis reveals distinct flavonoid biosynthesis regulation for leaf color development of *Cymbidium sinense* ‘Red Sun’. Int J Mol Sci. 2020;21:1869. 32182912 10.3390/ijms21051869 PMC7084835

[166] Lim GH , Kim SW, Ryu J et al. Upregulation of the MYB2 transcription factor is associated with increased accumulation of anthocyanin in the leaves of *Dendrobium bigibbum*. Int J Mol Sci. 2020;21:5653. 32781758 10.3390/ijms21165653 PMC7460623

[167] Li D , Yin H, Zhao C et al. Transcriptome analysis of tessellated and green leaves in *Paphiopedilum* orchids using Illumina paired-end sequencing and discovery simple sequence repeat markers. J Plant Biochem Physiol. 2014;2:4.

[168] Tsai CC , Wu YJ, Sheue CR et al. Molecular basis underlying leaf variegation of a moth orchid mutant (*Phalaenopsis aphrodite* subsp. *formosana)*. Plant Sci. 2017;8:1333. 10.3389/fpls.2017.01333 PMC5529386 28798769

[169] Hsiao YY , Pan ZJ, Hsu CC et al. Research on orchid biology and biotechnology. Plant Cell Physiol. 2011;52:1467–86. 21791545 10.1093/pcp/pcr100

[170] Su WR , Chen WS, Koshioka M et al. Changes in gibberellin levels in the flowering shoot of *Phalaenopsis hybrida* under high temperature conditionswhen flower development is blocked. Plant Physiol Biochem. 2001;39:45–50.

[171] Wang WY , Chen WS, Chen WH et al. Influence of abscisic acid on flowering in *Phalaenopsis hybrida*. Plant Physiol Biochem. 2002;40:97–100.

[172] Li Y , Zhang B, Yu H. Molecular genetic insights into orchid reproductive development. J Exp Bot. 2022;73:1841–52. 35104310 10.1093/jxb/erac016

[173] Sawettalake N , Bunnag S, Wang Y et al. *DOAP1* promotes flowering in the orchid *Dendrobium* Chao Praya smile. Front Plant Sci. 2017;8:400. 28386268 10.3389/fpls.2017.00400 PMC5362595

[174] Jang S . Functional characterization of *PhapLEAFY*, a *FLORICAULA/LEAFY* ortholog in *Phalaenopsis aphrodite*. Plant Cell Physiol. 2015;56:2234–47. 26493518 10.1093/pcp/pcv130

[175] Hou CJ , Yang CH. Functional analysis of *FT* and *TFL1* orthologs from orchid (*Oncidium* Gower Ramsey) that regulate the vegetative to reproductive transition. Plant Cell Physiol. 2009;50:1544–57. 19570813 10.1093/pcp/pcp099

[176] Wang SL , Viswanath KK, Tong CG et al. Floral induction and flower development of orchids. Front Plant Sci. 2019;10:1258. 31649713 10.3389/fpls.2019.01258 PMC6795766

[177] Xiang L , Li X, Qin D et al. Functional analysis of *FLOWERING LOCUS T *orthologs from spring orchid (*Cymbidium goeringii* Rchb. F.) that regulates the vegetative to reproductive transition. Plant Physiol Biochem. 2012;58:98–105. 22796899 10.1016/j.plaphy.2012.06.011

[178] Zhou S , Jiang L, Guan S et al. Expression profiles of five *FT*-like genes and functional analysis of *PhFT-1* in a *Phalaenopsis* hybrid. Electron J Biotechnol. 2018;31:75–83.

[179] Ke YT , Lin KF, Gu CH et al. Molecular characterization and expression profile of *PaCOL1*, a *CONSTANS*-like gene in *Phalaenopsis* orchid. Plan Theory. 2020;9:68. 10.3390/plants9010068 PMC7020484 31947959

[180] Ding L , Wang Y, Yu H. Overexpression of *DOSOC1*, an ortholog of *Arabidopsis**SOC1*, promotes flowering in the orchid *Dendrobium* Chao Parya smile. Plant Cell Physiol. 2013;54:595–608. 23396600 10.1093/pcp/pct026

[181] Chang YY , Chiu YF, Wu JW et al. Four orchid (*Oncidium* Gower Ramsey) *AP1*/*AGL9*-like MADS box genes show novel expression patterns and cause different effects on floral transition and formation in *Arabidopsis thaliana*. Plant Cell Physiol. 2009;50:1425–38. 19541596 10.1093/pcp/pcp087

[182] Jiang L , Jiang X, Li Y et al. *FT*-like paralogs are repressed by an SVP protein during the floral transition in *Phalaenopsis* orchid. Plant Cell Rep. 2022;41:233–48. 34713321 10.1007/s00299-021-02805-2

[183] Li Y , Zhang B, Wang Y et al. *DOTFL1* affects the floral transition in orchid *Dendrobium* Chao Praya Smile. Plant Physiol. 2021;186:2021–36. 33930147 10.1093/plphys/kiab200 PMC8331145

[184] Tsai WC , Kuoh CS, Chuang MH et al. Four DEF-like MADS box genes displayed distinct floral morphogenetic roles in *Phalaenopsis* orchid. Plant Cell Physiol. 2004;45:831–44. 15295066 10.1093/pcp/pch095

[185] Ahmad S , Lu C, Wei Y et al. The *de novo* transcriptome identifies important zinc finger signatures associated with flowering in the orchid *Arundina graminifolia*. Sci Hortic. 2022;291:110572.

[186] An FM , Hsiao SR, Chan MT. Sequencing-based approaches reveal low ambient temperature-responsive and tissue-specific microRNAs in *Phalaenopsis* orchid. PLoS One. 2011;6:e18937. 21573107 10.1371/journal.pone.0018937 PMC3089612

[187] Aceto S , Sica M, Paolo S et al. The analysis of the inflorescence miRNome of the orchid *Orchis italica* reveals a *DEF*-like MADS-box gene as a new miRNA target. PLoS One. 2014;9:e97839. 24832004 10.1371/journal.pone.0097839 PMC4022656

[188] Lin CS , Chen JJ, Huang YT et al. Catalog of *Erycina pusilla* miRNA and categorization of reproductive phase-related miRNAs and their target gene families. Plant Mol Biol. 2013;82:193–204. 23575662 10.1007/s11103-013-0055-y

[189] Ren Z , Ji X, Jiao Z et al. Functional analysis of a novel C-glycosyltransferase in the orchid *Dendrobium catenatum*. Hortic Res. 2020;7:111. 32637139 10.1038/s41438-020-0330-4 PMC7326982

[190] Kunst L , Samuels L. Plant cuticles shine: advances in wax biosynthesis and export. Curr Opin Plant Biol. 2009;12:721–7. 19864175 10.1016/j.pbi.2009.09.009

[191] Lu HC , Lam SH, Zhang D et al. R2R3-MYB genes coordinate conical cell development and cuticular wax biosynthesis in *Phalaenopsis aphrodite*. Plant Physiol. 2022;188:318–31. 34618124 10.1093/plphys/kiab422 PMC8774817

[192] Liu H , Wang L, Jing X et al. Functional analysis of *CgWRKY57* from *Cymbidium goeringii* in ABA response. PeerJ. 2021;9:e10982. 33665039 10.7717/peerj.10982 PMC7908890

[193] Xu Z , Li F, Li M et al. Functional analysis of ARF1 from *Cymbidium goeringii* in IAA response during leaf development. PeerJ. 2022;10:e10982. 10.7717/peerj.13077 PMC8918147 35291484

[194] Teoh ES . Medicinal orchids of Central America. In: Teoh ES, ed. Orchids as Aphrodisiac, Medicine or Food. Springer: Switzerland, 2019,139–58.

[195] Groves M , Rutherford C. Case studies and overviews of selected key orchid species in international commerce. Report to the 23rd Meeting of the CITES Plants Committee, Geneva, 2017. https://cites.org/sites/default/files/eng/com/pc/23/E-PC23-32.pdf.

[196] Park SY , Huh YS, Paek KY. Common protocols in orchid micropropagation. In: Lee YI, Yeung ET. (eds), Orchid Propagation: From Laboratories to Greenhouses—Methods and Protocols. Humana Press: New York, 2018, 179–93.

[197] Yam TW , Arditti J. Orchid micropropagation: an overview of approaches and methodologies. In: Lee YI, Yeung ET, eds. *Orchid Propagation: From Laboratories to Greenhouses—Methods and Protocols*. Springer Protocols Handbooks. Humana Press: New York, 2018,151–78.

[198] Bhattacharyya P , Kumaria S, Tandon P. High frequency regeneration protocol for *Dendrobium nobile*: a model tissue culture approach for propagation of medicinally important orchid species. S Afr J Bot. 2016;104:232–43.

[199] David D , Rusdi NA, Mohd Mokhtar RA et al. Establishment of in vitro regeneration protocol for Sabah’s Jewel orchid, *Macodes limii* J.J. wood & a.L. lamb. Horticulturae. 2022;8:155.

[200] Bhattacharyya P , Kumaria S, Job N et al. Phyto-molecular profiling and assessment of antioxidant activity within micropropagated plants of *Dendrobium thyrsiflorum*: a threatened, medicinal orchid. Plant Cell Tissue Organ Cult. 2015;122:535–50.

[201] Tikendra L , Amom T, Nongdam P. Molecular genetic homogeneity assessment of micropropagated *Dendrobium moschatum* Sw.-a rare medicinal orchid, using RAPD and ISSR markers. Plant Gene. 2019;19:100196.

[202] Longchar TB , Deb CR. Optimization of *in vitro* propagation protocol of *Dendrobium heterocarpum* wall. Ex. Lindl. and clonal genetic fidelity assessment of the regenerates: An orchid of horticultural and medicinal importance. S Afr J Bot. 2022;149:67–78.

[203] Teixeira da Silva JA , Cardoso JC, Dobránszki J et al. *Dendrobium* micropropagation: a review. Plant Cell Rep. 2015;34:671–704. 26046143 10.1007/s00299-015-1754-4

[204] Ahmad S , Chen J, Chen G et al. Transcriptional proposition for uniquely developed protocorm flowering in three orchid species: resources for innovative breeding. Front Plant Sci. 2022;13:942591. 35837448 10.3389/fpls.2022.942591 PMC9275812

[205] Yeow LC , Chew BL, Sreeramanan S et al. Elevation of secondary metabolites production through light-emitting diodes (LEDs) illumination in protocorm-like bodies (PLBs) of *Dendrobium* hybrid orchid rich in phytochemicals with therapeutic effects. Biotechnol Rep. 2020;27:e00497. 10.1016/j.btre.2020.e00497 PMC7365977 32695616

[206] Paul P , Kumaria S. Precursor-induced bioaccumulation of secondary metabolites and antioxidant activity in suspension cultures of *Dendrobium fimbriatum*, an orchid of therapeutic importance. S Afr J Bot. 2020;135:137–43.

[207] Singh N , Kumaria S. Deciphering the role of stress elicitors on the differential modulation of chalcone synthase gene and subsequent production of secondary metabolites in micropropagated *Coelogyne ovalis* Lindl., a therapeutically important medicinal orchid. S Afr J Bot. 2021;140:336–48.

[208] Mii M , Chin DP. Genetic transformation on orchid species: an overview of approaches and methodologies. In: Lee YI, Yeung ET, eds. *Orchid Propagation: From Laboratories to Greenhouses—Methods and Protocols*. Springer Protocols Handbooks. Humana Press: New York, 2018,151–65.

[209] Gaj T , Gersbach CA, Barbas CF. ZFN, TALEN, and CRISPR/Cas-based methods for genome engineering. Trends Biotechnol. 2013;31:397–405. 23664777 10.1016/j.tibtech.2013.04.004 PMC3694601

[210] Yin K , Gao C, Qiu JL. Progress and prospects in plant genome editing. Nat Plants. 2017;3:17107. 28758991 10.1038/nplants.2017.107

[211] McManus MT , Sharp PA. Gene silencing in mammals by small interfering RNAs. Nat Rev Genet. 2002;3:737–47. 12360232 10.1038/nrg908

[212] Ratanasut K , Monmai C, Piluk P. Transient hairpin RNAi-induced silencing in floral tissues of *Dendrobium* Sonia ‘Earsakul’ by agroinfiltration for rapid assay of flower colour modification. Plant Cell Tissue Organ Cult. 2015;120:643–54.

[213] Liu JX , Chiou CY, Shen CH et al. RNA interference-based gene silencing of phytoene synthase impairs growth, carotenoids, and plastid phenotype in *Oncidium hybrid* orchid. Springerplus. 2014;3:212. 25221736 10.1186/2193-1801-3-478 PMC4161717

[214] Liu YC , Yeh CW, Der CJ et al. Petal-specific RNAi-mediated silencing of the phytoene synthase gene reduces xanthophyll levels to generate new *Oncidium* orchid varieties with white-colour blooms. Plant Biotechnol J. 2019;17:2035–7. 31145513 10.1111/pbi.13179 PMC6790357

[215] Liu X , Wu S, Xu J et al. Application of CRISPR/Cas9 in plant biology. Acta Pharm Sin B. 2017;7:292–302. 28589077 10.1016/j.apsb.2017.01.002 PMC5443236

[216] Kui L , Chen H, Zhang W et al. Building a genetic manipulation tool box for orchid biology: identification of constitutive promoters and application of CRISPR/Cas9 in the orchid, *Dendrobium officinale*. Front Plant Sci. 2017;7:2036. 28127299 10.3389/fpls.2016.02036 PMC5226938

[217] Tong CG , Wu FH, Yuan YH et al. High-efficiency CRISPR/Cas-based editing of *Phalaenopsis* orchid MADS genes. Plant Biotechnol J. 2020;18:889–91. 31553827 10.1111/pbi.13264 PMC7061860

[218] Semiarti E , Nopitasari S, Setiawati Y et al. Application of CRISPR/Cas9 genome editing system for molecular breeding of orchids. Indones J Biotechnol. 2020;25:61–8.

[219] Li Y , Zhang B, Yu H. Kilobase-scale genomic deletion of *DOTFL1* in *Dendrobium* orchids. J Genet Genomics. 2022;49:81–4. 34426098 10.1016/j.jgg.2021.07.008

[220] Soda N , Verma L, Giri J. CRISPR-Cas9 based plant genome editing: significance, opportunities and recent advances. Plant Physiol Biochem. 2018;131:2–11. 29103811 10.1016/j.plaphy.2017.10.024

[221] Zhang W , Zhang G, Zeng P et al. Genome sequence of *Apostasia ramifera* provides insights into the adaptive evolution in orchids. BMC Genomics 2021;22:536. 10.1186/s12864-021-07852-3 PMC8278605 34256691

[222] Bae EK , An C, Kang MJ et al. Chromosome-level genome assembly of the fully mycoheterotrophic orchid *Gastrodia elata*. G3 Genes|Genomes|Genetics. 2022;12:jkab433. 35100375 10.1093/g3journal/jkab433 PMC8896018

[223] Han B , Jing Y, Dai J et al. A chromosome-level genome assembly of *Dendrobium huoshanense* using long reads and Hi-C data. Genome Biol Evol. 2020;12:2486–90. 33045048 10.1093/gbe/evaa215 PMC7846097

[224] Chung O , Kim J, Bolser D et al. A chromosome-scale genome assembly and annotation of the spring orchid (*Cymbidium goeringii*). Mol Ecol Resources. 2022;2022:1168–77. 10.1111/1755-0998.13537 34687590

[225] Liao LJ , Pan IC, Chan YL et al. Transgene silencing in *Phalaenopsis* expressing the coat protein of *Cymbidium* mosaic virus is a manifestation of RNA-mediated resistance. Mol Breed. 2004;13:229–42.

[226] Chan YL , Lin KH, Sanjaya et al. Gene stacking in *Phalaenopsis* orchid enhances dual tolerance to pathogen attack. Transgenic Res. 2005;14:279–88. 16145836 10.1007/s11248-005-0106-5

[227] Qin X , Liu Y, Mao S et al. Genetic transformation of lipid transfer protein encoding gene in *Phalaenopsis amabilis* to enhance cold resistance. Euphytica. 2011;177:33–43.

[228] Liang CY , Rengasamy KP, Huang LM et al. Assessment of violet-blue color formation in *Phalaenopsis* orchids. BMC Plant Biol. 2020;20:212. 32397954 10.1186/s12870-020-02402-7 PMC7218627

[229] Hsieh KT , Liu SH, Wang IW et al. *Phalaenopsis* orchid miniaturization by overexpression of *OsGA2ox6*, a rice GA2-oxidase gene. Bot Stud. 2020;61:1–11. 32253516 10.1186/s40529-020-00288-0 PMC7136379

[230] Chang L , Tzean Y, Hsin KT et al. Stress associated proteins coordinate the activation of comprehensive antiviral immunity in *Phalaenopsis* orchids. New Phytol. 2022;233:145–55. 34614215 10.1111/nph.17776

[231] Lee SH , Li CW, Liau CH et al. Establishment of an *agrobacterium*-mediated genetic transformation procedure for the experimental model orchid *Erycina pusilla*. Plant Cell Tissue Organ Cult. 2015;120:211–20.

[232] Yu H , Yang SH, Goh CJ. *Agrobacterium*-mediated transformation of a *Dendrobium* orchid with the class 1 *knox* gene *DOH1*. Plant Cell Rep. 2001;20:301–5.

[233] Petchthai U , Chuphrom A, Huehne PS. Recovery of virus-infected *Dendrobium* orchids by constitutive expression of the *Cymbidium* mosaic virus coat protein gene. Plant Cell Tissue Organ Cult. 2015;120:597–606.

[234] Wang Y , Liu L, Song S et al. *DOFT* and *DOFTIP1* affect reproductive development in the orchid *Dendrobium* Chao Praya smile. J Exp Bot. 2017;68:5759–72. 29186512 10.1093/jxb/erx400 PMC5854133

[235] Sornchai P , van Doorn WG, Imsabai W et al. *Dendrobium* orchids carrying antisense ACC oxidase: small changes in flower morphology and a delay of bud abortion, flower senescence, and abscission of flowers. Transgenic Res. 2020;29:429–42. 32691287 10.1007/s11248-020-00209-8

[236] Raffeiner B , Serek M, Winkelmann T. Agrobacterium tumefaciens-mediated transformation of *Oncidium* and *Odontoglossum* orchid species with the ethylene receptor mutant gene *etr1-1*. Plant Cell Tissue Organ Cult. 2009;98:125–34.

[237] Thiruvengadam M , Chung IM, Yang CH. Overexpression of *Oncidium* MADS box (*OMADS1*) gene promotes early flowering in transgenic orchid (*Oncidium* Gower Ramsey). Acta Physiol Plant. 2012;34:1295–302.

